# Brilliant structural colors originating from reflection by nanogaps of nacreous layers in fossilized ammonite shells

**DOI:** 10.1038/s41598-025-21872-z

**Published:** 2025-10-30

**Authors:** Naoki Hizukuri, Yuri Oshima, Yuta Yagi, Hayato Sasazawa, Yuya Oaki, Hiroyuki Tsuda, Hiroaki Imai

**Affiliations:** School of Integrated Design Engineering, Faculty of Science and Technology, Keio University, 3-14-1 Hiyoshi, Kohoku-ku, Yokohama, 223-8522 Japan

**Keywords:** Materials science, Optics and photonics

## Abstract

Vivid-colored ammonite fossils that are mined in Alberta, Canada, are known as gemstones called “Ammolite.” The specific colors having narrow reflectance spectra are ascribed to an interference phenomenon on the preserved nacre of fossils. However, the essence of the vivid tones that are different from the structural coloration of other kinds of shells has not been revealed. In this work, we clarified that specific reflection by interlaminar nanogaps (~ 4 nm) of aragonite plates with the homogeneous lamination period in the fossilized nacreous layers is essential for the emergence of vivid structural colors depending on the thickness of the layer.

**Supplementary Information:**

The online version contains supplementary material available at 10.1038/s41598-025-21872-z.

## Introduction

Structural coloration is the color produced by interfering with visible light by periodic microstructures, as in reflection, diffraction, and scattering^1–4^. Fadeless color and iridescence, which is a change of color when viewed from different angle, are unique characteristics of structural coloration. In nature, we observe structural colors on many bird feathers and butterfly wings^5–10^. The nacreous layer of seashells and the coleoptera of jewel beetles show structural colors originating from multilayer interference due to microscopically layered structures^11,12^. In general, colors caused by chitin layers of the coleoptera seem to be vivid because their saturation is higher than that of the nacreous layers of modern shells. Interestingly, a kind of preserved nacre in fossils shows brilliant colors similar to those of the coleopteran, such as an elytron of a jewel beetle (*Chrysochroa fulgidissimaa*) (Fig. S1a–c in the supporting information (SI)). Moreover, the reflectance spectrum of the fossil narrower than that of the coleoptera indicate the feature of the vivid tone (Fig. S1d in the SI). However, the origin of this fascinating structural color of the fossils has not been clarified with detailed characterization. In this study, we focus on the particular colors of the fossilized nacreous layers that mimic nacreous layers of shells^13–16^ bright structural coloration originating from multilayer interference.

Nacreous layers of shells^13–16^ from many Mollusca are composed of platelet aragonite crystals and small amounts of organic matter. Aragonite platelets in the nacreous layers expose a large (001) plane, and organic thin sheets exist between the plates. The interlamellar sheets were reported to be mainly composed of chitin and proteins in previous works^17–19^. Since aragonite crystals have refractive indexes (*n* = 1.53 across the *c* axis, *n* = 1.686 across the *b* axis, and *n* = 1.681 across the *a* axis^20^ that are higher than that of organic sheets^11^, light incident to the nacreous layers is reflected at the boundary between the crystal plates and the organic sheets. The nacreous layers show pearly luster and specific colors^11,15^ originating from multilayer interference through the interaction of reflecting lights.

**Fig. 1:**
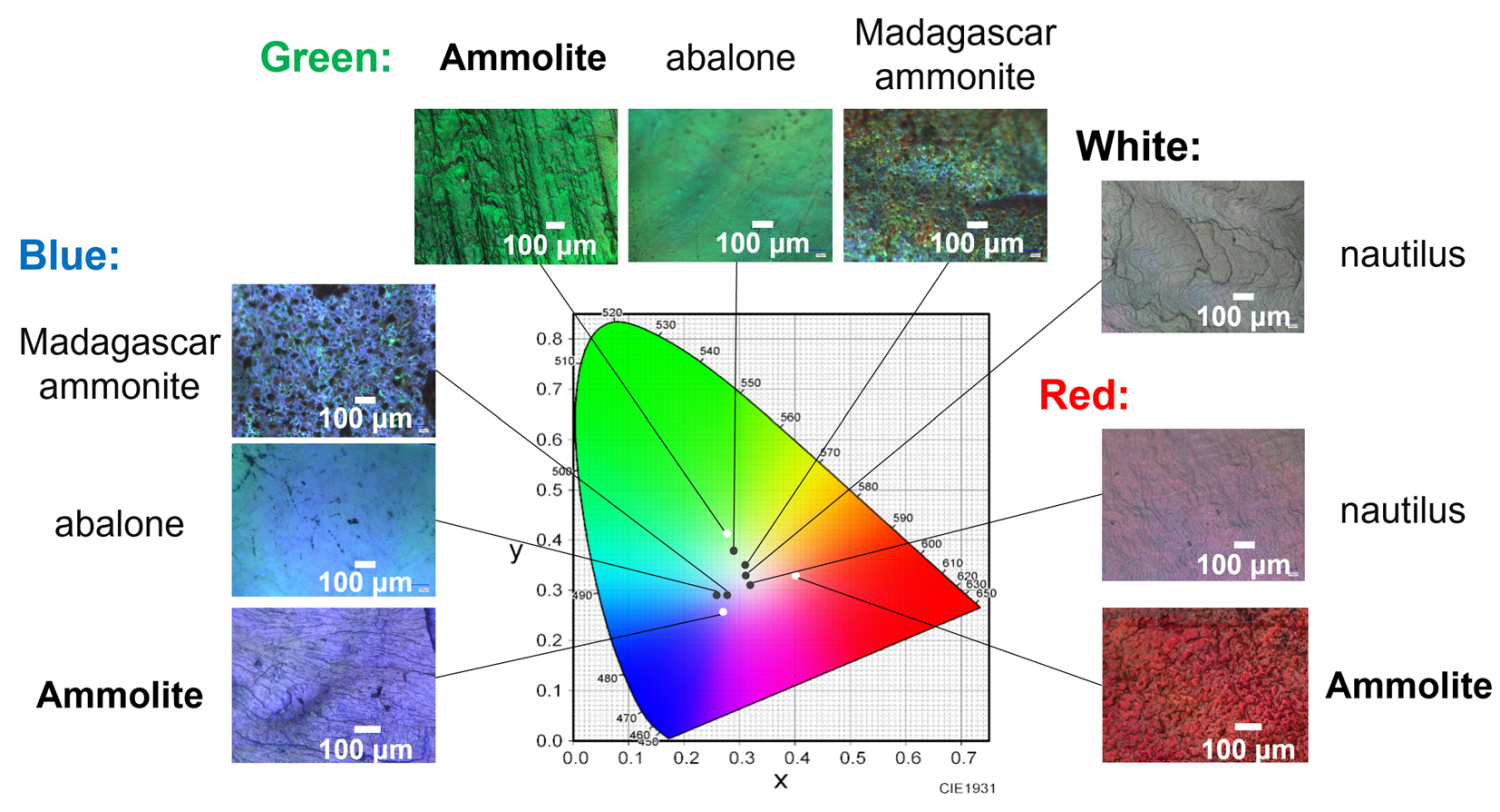
Photographs of brilliantly colored pieces (blue, green, and red) of Ammolite, shells of abalones and nautilus, and an ammonite fossil mined in Madagascar with a color map on the Commission Internationale de I’Edairage (CIE) chromaticity diagram. The central region and edges of the diagram indicate low and high color saturations, respectively. The structural colors were evaluated quantitatively from photographs using an image analysis software (ImageJ Fiji). The values of x, y, and z (x + y + z = 1) were calculated from the three primary colors.

Ammonites were a group of extinct marine cephalopods having squid-like organisms that were particularly abundant during the Jurassic and Cretaceous periods (about 200–65 million years ago). Disk-shaped coiled shells of ammonites are divided internally into several chambers. When the nacreous layer consisting of aragonite plates is preserved in fossilized ammonite shells, we observe iridescent structural coloration similar to that of modern shells^21^. Particularly, Ammolite, which is a gemstone trade name, is known as brilliantly colored shells of extinct species—such as *Placenticeras meeki*, *Placenticeras costatum*, and *Placenticeras intercalare—*that were mined near the St. Mary River in Alberta, Canada^22^. Vibrant red, green, and blue pieces of Ammolite are shown with other kinds of shells in Figs. 1 and S2 in the SI. Whereas pale colors and pearly luster are observed on abalone and nautilus shells, respectively, the surface of Ammolite shows brilliant colors, such as clear red, bright green, and deep blue. The color saturation of Ammolite seems to be higher than that of the others. While abundant fossil ammonites are distributed all over the world, brilliantly colored fossils have been obtained from only a few localities, such as the Bearpaw Formation of the Late Cretaceous age (about 75–73 million years ago)^22^. Thus, the production of brilliant colors would require the modification of the nacreous layer under particular conditions. Although the coloration of Ammolite is ascribed to the multilayer interference of the laminated structures^2^, the origin of brilliant colors has not been studied based on experimental evidence.

In this study, we characterized the microstructure and optical properties of nacreous layers in various colored pieces of Ammolite to reveal the structural features and the origin of the exhibition of brilliant colors. The nacreous layers of abalone and nautilus shells and ammonite fossils mined in Madagascar were compared as references (Figs. 1 and S2d–f in the SI). The origin of vivid colors was studied with the characterization of laminated structures consisting of aragonite plates with particular optical properties using optical simulation^23^. Finally, we found that specific reflection by interlaminar nanogaps (~ 4 nm) of aragonite plates with the homogeneous lamination period in the nacreous layers is essential factors for the emergence of the brilliant colors of the Ammolite. The specific structural color and its mechanism would be utilized in various industrial applications, such as non-fading color coatings.

## Results and discussion

### Optical properties

Figure 1 shows the Commission Internationale de I’Edairage (CIE) chromaticity diagram, which allows the comparison of hues and saturation by mapping colors visible to the human eye. The x and y values of colors for the nacreous layers of abalone and nautilus shells and a Madagascar ammonite fossil are located near the center of the diagram. This means that their colors are pale with low chroma. On the other hand, the nacreous layers of the Ammolite are found to show vivid monochromatic colors, such as red, green, and blue. In these cases, their x and y values are located in regions relatively near the edges of the red, green, and blue regions. Thus, we can evaluate the color saturation (chroma) quantitatively from the diagram. As shown in Fig. S3 in the SI, powders obtained by the grinding of several pieces of a fossil were pale brown regardless of the original color. This supports the contention that the coloration of the Ammolite originates from its structures, whereas the embrowning of the powders is attributable to a small amount of organic impurities (< 2 wt%) (Fig. S4 in the SI).

**Fig. 2:**
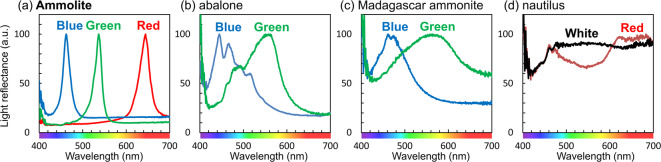
Reflectance spectra from various nacreous layers. (**a**) Red, green, and blue pieces of Ammolite; (**b**) blue and green parts of an abalone shell; (**c**) blue and green parts of the Madagascar ammonite fossil; and (**d**) red and white parts of the nacres of a nautilus shell. Reflectance spectra were measured with the experimental setup shown in Fig. S6 in the SI. Light was incident vertically from the light source (*θ* = 0°).

We characterized the colors of nacreous layers using reflectance spectra, as shown in Figs. 2, S1 and S5 in the SI. Brilliant colors—such as clear red, bright green, and deep blue—of the Ammolite provide sharp reflection bands centered at 640, 540, and 460 nm, respectively. On the other hand, the reflectance bands for the green elytron of the jewel beetle and the blue and green parts of the Madagascar ammonite fossil and abalone shells are broadened with white noise in all of the visible region. A distinct reflection around a specific wavelength was not observed on the nacres of a nautilus shell. In a previous work, similar refraction was reported for abalone shells with experimental data and theoretical simulation^11^. These results indicate that brilliant colors from the nacreous layers can be ascribed to the intense, narrow reflection bands around a specific wavelength without white noise in the visible region.

We observed iridescence on all Ammolite colors using a setup shown in Fig. S6 in the SI. Figure S7 in the SI shows the dependence of the color on the angle of incidence of light for red, green, and blue pieces of Ammolite. However, the angle dependence of color seems to be relatively weak. We will discuss angle-dependent color variation using theoretical calculation in the following section.

### Microstructures of the nacreous layers

**Fig. 3:**
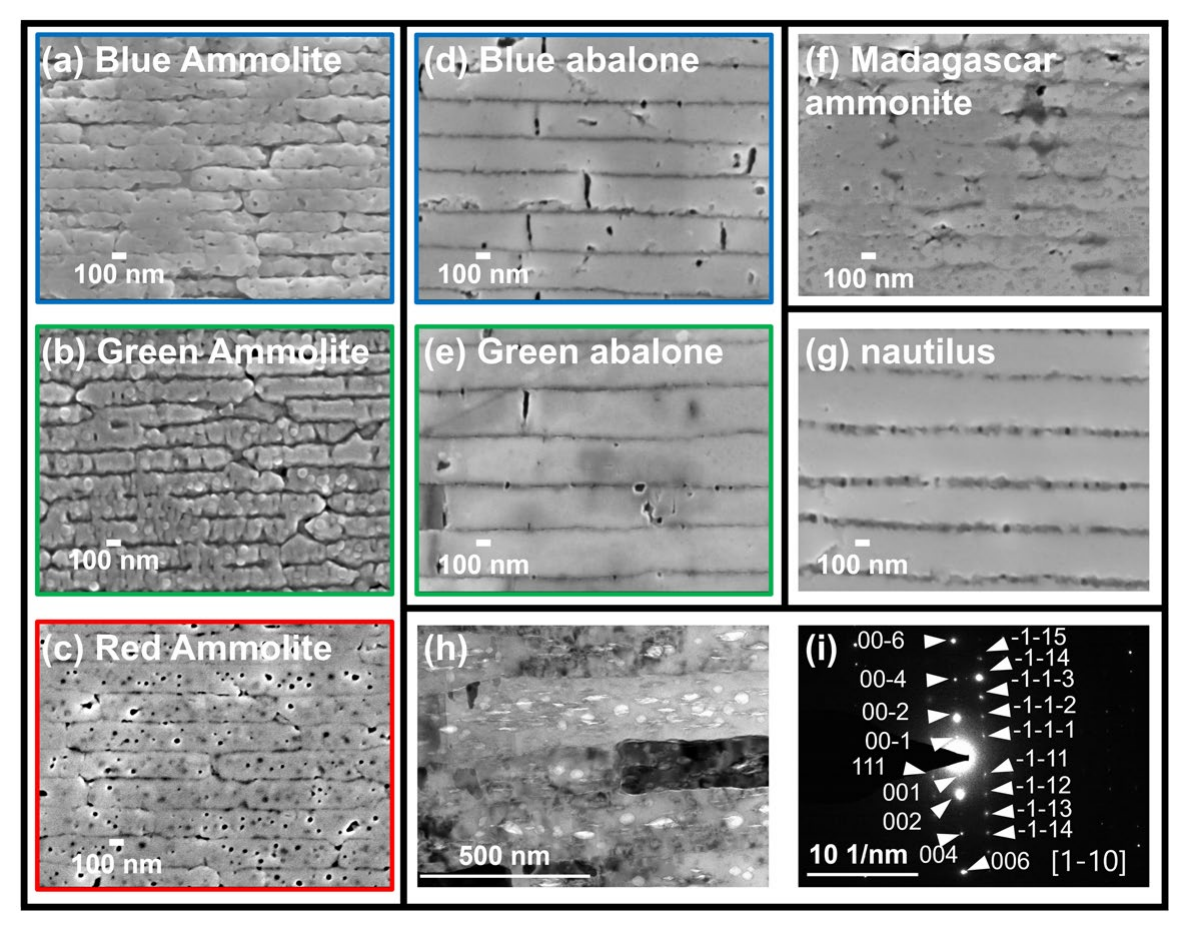
SEM images of a polished cross section of the nacreous layers. (**a**) A blue piece of Ammolite, (**b**) a green piece of Ammolite, (**c**) a red piece of Ammolite, (**d**) a blue part of abalone, (**e**) a green part of abalone, (**f**) a Madagascar ammonite fossil, and (**g**) a nautilus shell. (**h**) A cross-sectional TEM image and (**i**) an SAED pattern of an FIB-cut sample of the fossilized nacreous layer of a red piece of Ammolite.

From scanning electron microscopy (SEM) images of polished cross sections of the nacreous layers of Ammolites, abalones, and the Madagascar ammonite fossil (Fig. 3a–g), we observed the similarity of the laminated structures. We studied the crystallographic features of the fossilized nacres using electron diffraction. Panels h and i in Fig. 3 show cross-sectional images of transmission electron microscopy (TEM) and selected area electron diffraction (SAED) patterns of focused ion beam (FIB)–cut samples of nacreous layers of Ammolite, respectively. Aragonite plates that are stacked in the *c*-axis orientation are the same as those in contemporary shells. These results mean that the basic structure—consisting of aragonite plates—is preserved in the fossils.

**Fig. 4:**
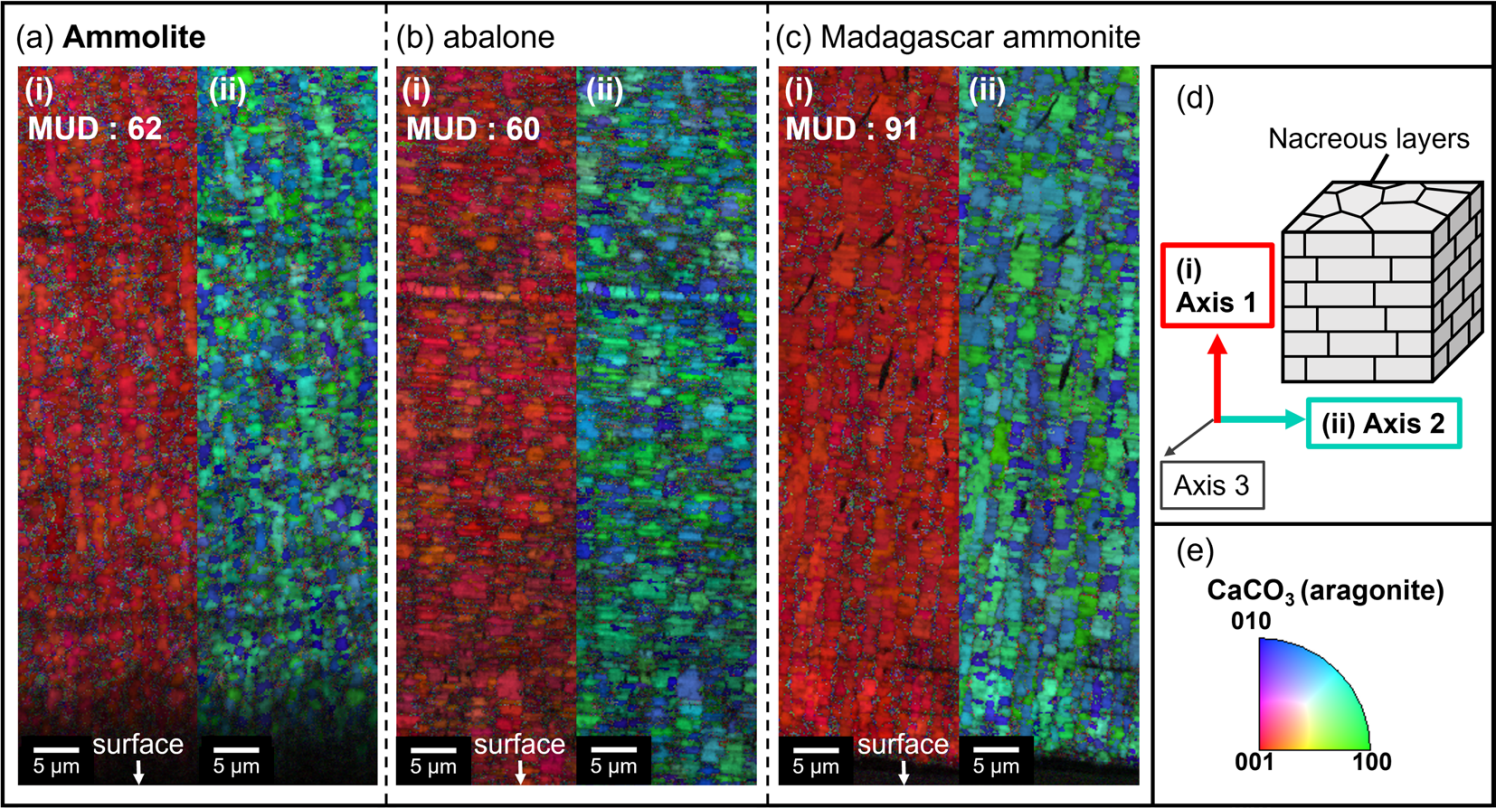
Inverse pole figure (IPF) maps of the EBSD scans for polished cross sections of the nacreous layers. (**a**) Ammolite (green), (**b**) abalone (blue), and (**c**) Madagascar ammonite (blue). The MUD values, which indicate the strength of crystal co-orientation^24^, were obtained from the pole figures (Fig. S8 in the SI). (**d**) Directions of Axis 1, Axis 2, and Axis 3 on a cross section of the nacreous layers. The IPF maps indicate the crystallographic orientations along (i) Axis 1 and (ii) Axis 2. (**e**) The EBSD color code of CaCO_3_ (aragonite). The red colored images for Axis 1 (i) mean that the *c* axis ([001]) is perpendicular to the surface of the nacreous layer. The mosaic images of green, blue, and pale blue for Axis 2 (ii) suggest that the *a* ([100]) and *b* ([010]) axes of aragonite tablets rotate along the *c* axis in the nacreous layer.

The crystallographic structure of various nacreous layers was also characterized by the electron backscatter diffraction (EBSD) as shown in Figs. 4 and S8 in the SI. The inverse pole figure (IPF) maps indicate that the nacreous layers of the fossils (Ammolite and Madagascar ammonite) are composed of the *c*-axis-oriented aragonite tablets which are similar to that of the abalone shell. Since the similarity of crystallographic orientation that was evaluated from color of the IPF maps with the multiple of uniform distribution (MUD) values between the modern shell and fossils, we conclude that diagenetic variation including overprint effects^24^ is not significant for the nacreous layers of the Ammolite and Madagascar ammonite. Here, the differences in coloration between the samples cannot be explained by the crystallographic feature of the nacreous layers.

**Fig. 5:**
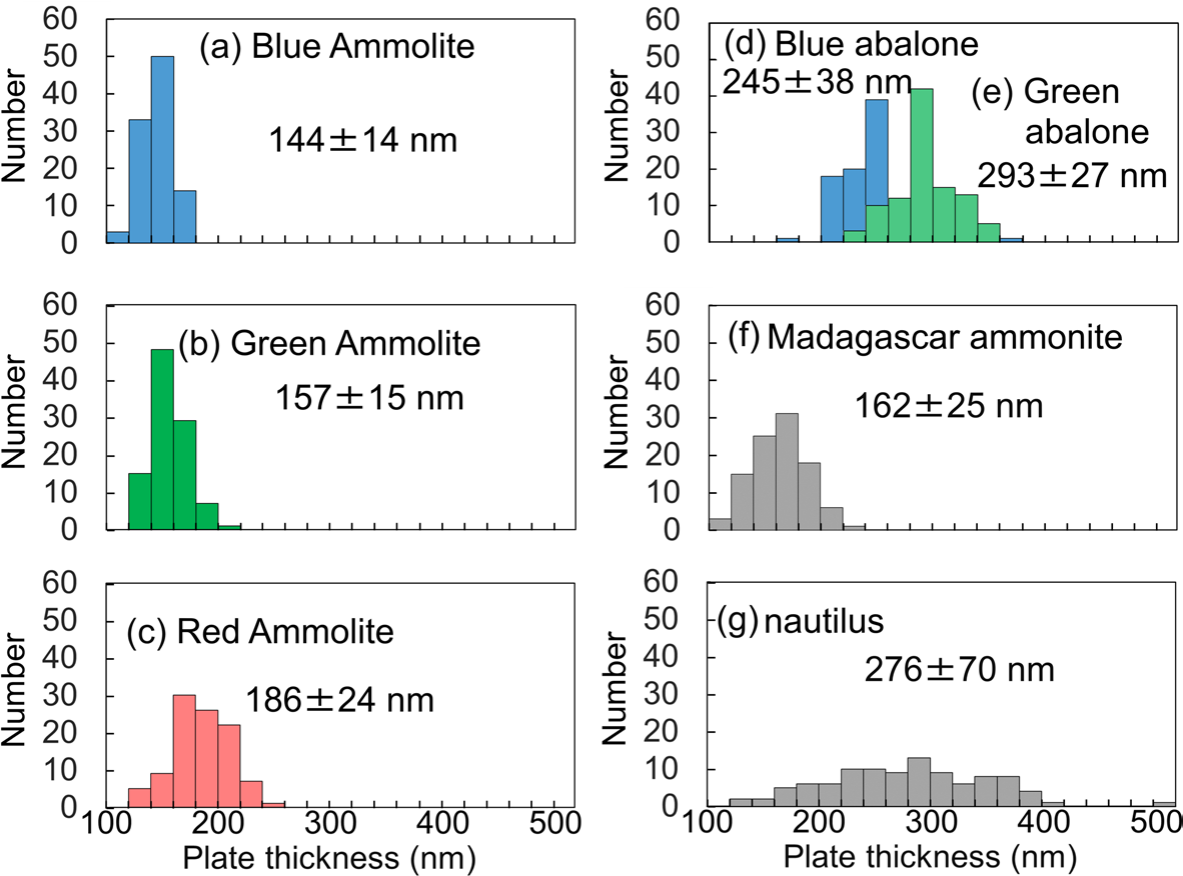
Distribution of aragonite plate thickness (*d*_1_) in the nacreous layers. (**a**) A blue piece of Ammolite, (**b**) a green piece of Ammolite, (**c**) a red piece of Ammolite, (**d**) a blue part of abalone, (**e**) a green part of abalone, (**f**) a Madagascar ammonite fossil, and (**g**) a nautilus shell. The average values of *d*_1_ with the standard deviation were shown in the panels.

The nacreous layers can be represented by a model with alternating layers of aragonite plates and interlaminar gaps (Fig. S9 in the SI). We compared the thickness of aragonite plates (*d*_1_) in the nacreous layers having various colors (Fig. 5). Basically, the color of the nacreous layer depends on the plate thickness in the laminated structures. Red, green, and blue pieces of Ammolite have plates with average thicknesses of 190, 160, and 140 nm, respectively. The dependence of the color on *d*_1_ is the same as reported in previous works^11,12,25,26^. The plates in the green part (*d*_1_ ∼300 nm) of abalone shell are thicker than those in the blue part (*d*_1_ ∼250 nm). Abalone plates are thicker than those of Ammolites. The relationship between *d*_1_ and color is discussed using the theoretical calculation and the optical simulation in the following section.

**Fig. 6:**
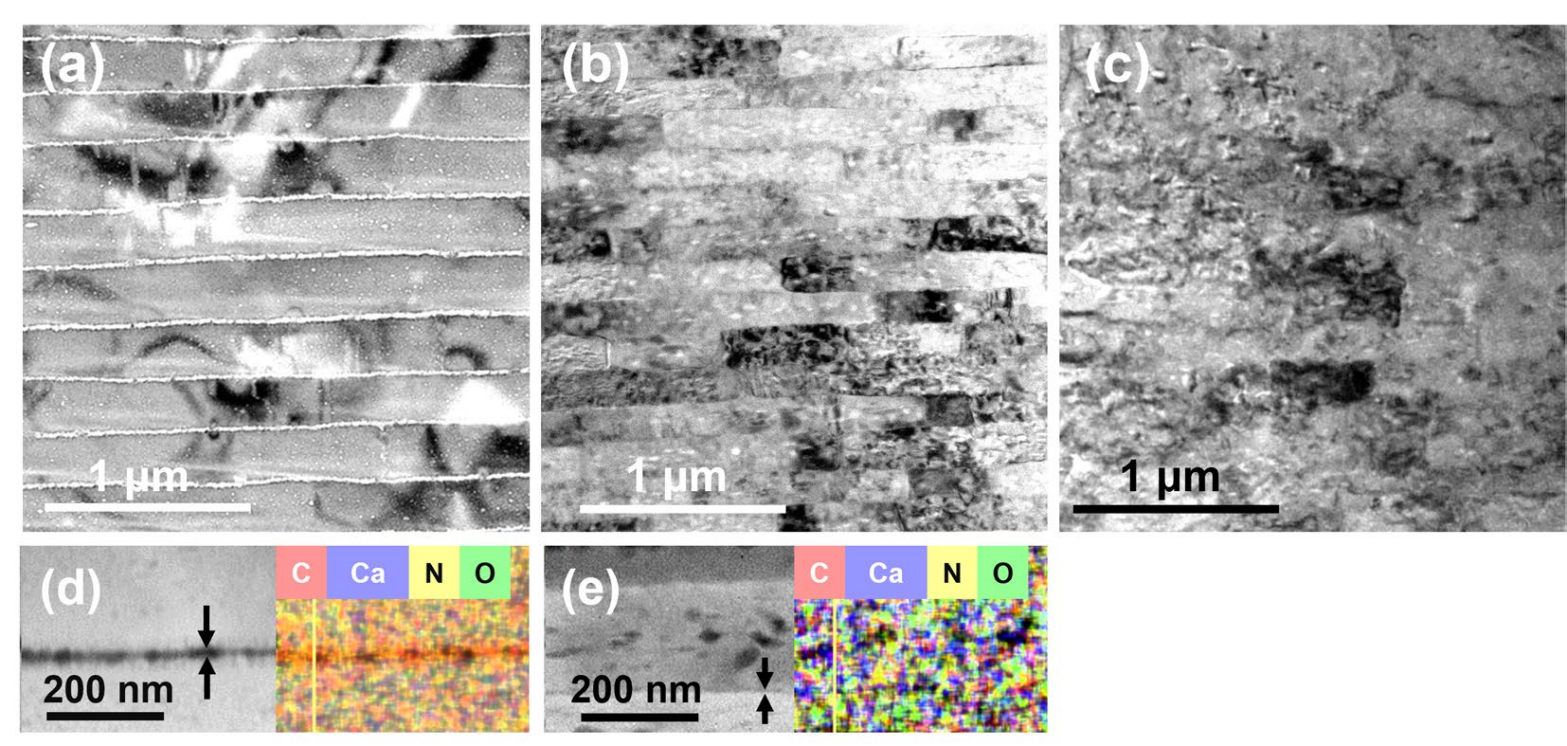
(**a**–**c**) TEM images with (**d**, **e**) EDS elemental mapping images of FIB-cut samples of the nacreous layers of abalone, Ammolite, and the Madagascar ammonite fossil. (**a**, **d**) An abalone, (**b**, **e**) a red piece of Ammolite, and (**c**) a Madagascar ammonite fossil.

We characterized the interlaminar gap distance (*d*_2_) between aragonite plates using TEM images of FIB-cut samples of nacreous layers of Ammolite, Madagascar ammonite fossil, and abalone (Fig. 6). The average value of *d*_2_ was 11 ± 3 nm in the nacreous layer of abalone with the presence of an organic sheet in the gap. The elemental mapping image (Fig. 6d) obtained by energy dispersive X-ray spectroscopy (EDS) indicates that the interlamellar gaps are filled by organic membranes. The presence of organic matter was also suggested by a weight loss in the range of 100–600 °C evaluated by thermogravimetric analysis (Fig. S4 in the SI). According to previous studies^17–19^, chitin and proteins are the main components of the organic membrane in the living mollusk. On the other hand, the interlaminar gaps of the Madagascar ammonite fossil collapsed in the laminated structure. We evaluated that the average value of *d*_2_ is 4 ± 2 nm, whereas some plates are directly attached to each other in the nacre of the Ammolite. Here, the organic sheets were not recognized in the elemental mapping image (Fig. 6e). The laminated architecture of the Ammolite is found to be the same as that of modern shells. However, small interlaminar gaps (nanogaps) are a feature of the Ammolite and are deduced to result in brilliant colors of the nacreous layers.

**Fig. 7:**
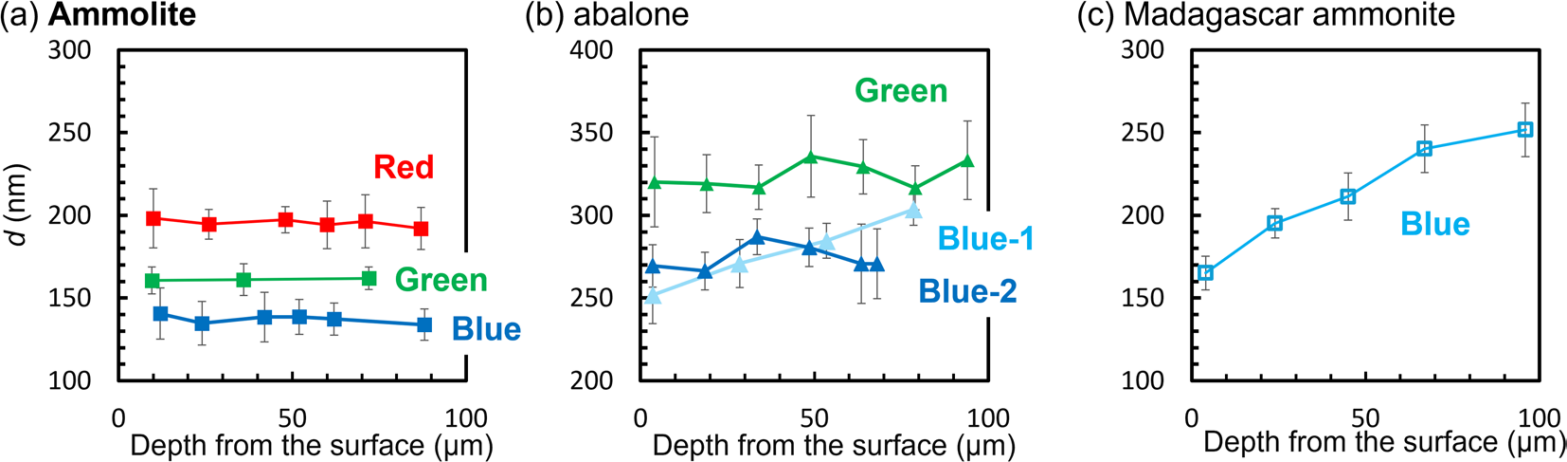
Depth profiles of the lamination periods (*d*). (**a**) Blue, green, and red pieces of Ammolite, (**b**) blue and green pieces of abalone shells, and (**c**) blue Madagascar ammonite.

The lamination period (*d*)—including *d*_1_ and *d*_2_—is essential for the structural color (Fig. S9 in the SI). We investigated the lamination periods in a wide range of nacreous layers for various samples (Figs. S10–S12 in the SI). Figure 7 shows the depth profiles of the lamination periods in the nacreous layers for blue, green, and red pieces of Ammolite, blue and green pieces of abalone shells, and blue Madagascar ammonite. The periods of blue, green, and red pieces of the Ammolite are relatively constant in the range of 100 μm, whereas those of the abalone shells and Madagascar ammonite are varied. This suggests that the appearance of brilliant colors requires the homogeneous periodicity of laminated structures in a certain range.

The lamination period is homogeneous over 400 μm for a high-quality green sample, whereas it is rapidly varied at 50 μm for a piece exhibiting pale orange (Figs. S11 and S12 in the SI). Thus, the homogeneity of the lamination period is related to the sample quality. The thickness of homogeneous periodicity required for brilliant colors is about 50 μm. The inhomogeneity of the lamination period causes a piece of Ammolite to change color with polishing. Figure S13 in the SI shows change of color from red Ammolite to green by polishing.

## Modification of nacreous layers of the abalone for vivid coloration

According to TEM observation (Fig. 6), nanogaps in the laminated structures are deduced to be essential for brilliant colors with high saturation. We verified the hypothesis by modifying the nacreous layer of abalone shells for vivid coloration as follows.

**Fig. 8:**
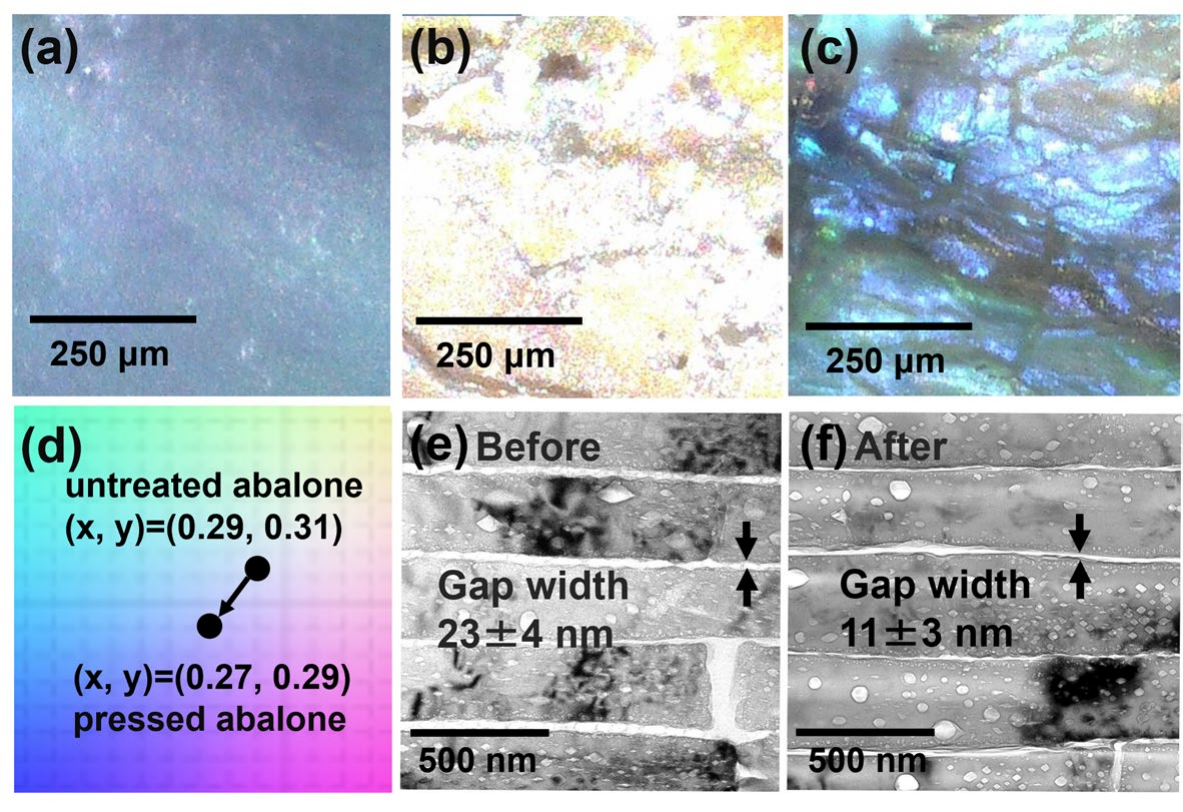
(**a**–**c**) Photos of the shell surface, (**d**) variation of x and y values in the CIE diagram, and (**e**, **f**) TEM images of FIB-cut samples of the nacreous layers of abalone shells (**a**, **e**) before treatment, (**b**) after removal of the organic matter, and (**c**, **f**) after subsequent pressing.

We removed organic substance between the nacreous layer of abalone shells and then decreased the interlaminar gaps under pressure. Figure 8 shows the color change of the abalone shell through a removal of the organic sheet and a decrease in the interplate gap under pressure. Abalone shells were treated in a sodium hydroxide solution to remove the organic sheets in the interlaminar gaps. The removal of the interlamellar organic matter was confirmed from the elemental mapping images by EDS (Fig. S14 in the SI). Abalone shells turned white with the removal of the organic matter (Fig. 8a, b). We then recovered the blue-green color after pressing at 1000 MPa (Fig. 8c). We confirmed the x and y values, and saturation increased after pressing (Fig. 8d).

The interlaminar gaps decreased from 23 to 11 nm with the exhibition of colors (Fig. 8e, f). The verification experiment with the characterization of the microstructures and optical properties is important for finding out the principle behind the high saturation for Ammolite. In this case, however, the lamination periodicity was not changed by the pressing experiment with the removal of the organic matter. Thus, brilliant color similar to that of the Ammolite was not perfectly achieved by the modification of the nacreous layer of abalone.

## Discussion of optical properties of the nacreous layers using theoretical calculation and simulation

We discussed the relationship between the lamination period and color using the Bragg-Snell equation that is expressed as Eq. (1) and Fig. S9 in the SI:

1 $$2d\sqrt{{n}^{2}-{\text{sin}}^{2}\theta }=m\lambda$$

where *d* is the lamination period, $\theta$ is the angle between the observer’s point of view and the lamella horizontal, *m* is the order of the Bragg-Snell reflection, and$\lambda$ is the wavelength of the light enhanced by multilayer refrection. The effective refractive index (*n*) is calculated by Eq. (2):

2 $$n=\sqrt{({n}_{1}^{2}{\varphi }_{1}+{n}_{2}^{2}{\varphi }_{2})}$$

where $\varphi$ stands for volume fraction of aragonite platelet $({\varphi }_{1}={d}_{1}/d)$ and gap$({\varphi }_{2}={d}_{2}/d)$. When $\theta \text{ is }$0°, the relationship between $d$ and $\lambda$ is calculated by Eq. (3):

3 $$2nd=m\lambda .$$

The refractive index of aragonite platelet was set to *n*_1_ = 1.63, which was used in a study on the optical properties of abalone^11^. The refractive index of the interlaminar gap was set in two ways: *n*_2_ = 1.43^11,27^ (organic layer for abalone) and *n*_2_ = 1.00 (air layer for Ammolite).

**Fig. 9:**
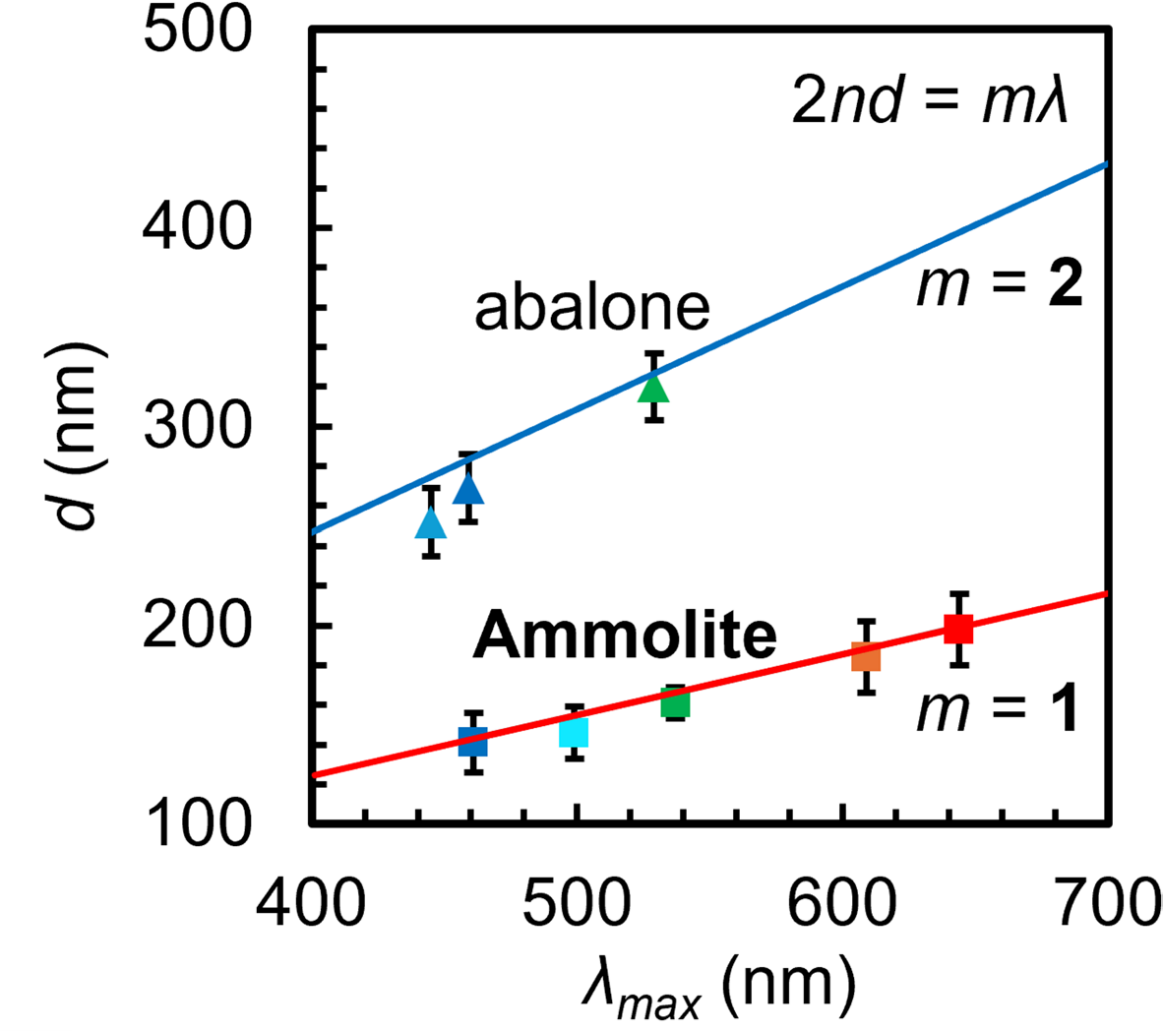
The relationship between the lamination period (*d*) and the peak wavelength (*λ*_max_) of the reflectance spectra for variously colored pieces of Ammolite (Fig. S15 in the SI) and abalone shells. The red and blue lines indicate the theoretically calculated values using Eq. (1) with *m* = 1 and 2, respectively.

Figures 9 and S15 in the SI show the relationship between *d* and the peak wavelength for variously colored pieces of Ammolite and abalone shells. The dependence of the peak wavelength on *d* agrees with the theoretical line for multilayer reflection. Thus, the coloration of Ammolite as well as abalone shells is commonly ascribed to the periodic structures of the nacres. The order of the reflectance (*m* = 1) for Ammolite is different from that (*m* = 2) of abalone. However, the appearance of brilliant colors cannot be explained by this calculation.

The angle dependence of the structural colors is explained by the theoretical calculation (Fig. S7a-iii, b-iii, c-iii in the SI). However, practically obtained peak wavelengths are shifted to positions longer than the calculated values. This weak angle dependence of the color may be ascribed to the disordered structure in the fossilized nacreous layers.

**Fig. 10:**
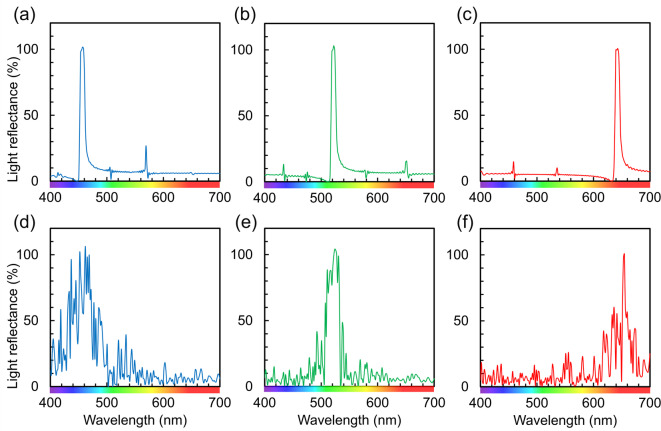
Simulated reflectance spectra from the periodic structures with plate thicknesses (*d*_1_) of (**a**, **d**) 137, (**b**, **e**) 157, and (**c**, **f**) 194 nm and an interlaminar gap (*d*_2_) 4 nm. The simulation was performed with (**d**) 18 nm, (**e**) 8 nm, and (**f**) 15 nm of deviation and without (**a**–**c**) a standard deviation of *d*_1_. Small peaks are ascribed to the higher order reflection. Noises in the signals may be due to variations in *d*_1_ of the model. The standard deviations were estimated from cross-sectional SEM images near the surface of blue, green, and red pieces of Ammolite (Fig. S15 in the SI).

We studied the structural colors of the nacreous layers using the two-dimensional (2D) finite-difference time-domain (FDTD) method using a 2D model^28–31^ (Fig. S16 in the SI). The reflectance spectra were simulated by changing the plate thickness (*d*_1_) and the gap width (*d*_2_) of the nacreous layers. In the first step, we evaluated the dependence of color on *d*_1_ and its variation. Here, *d*_2_ was fixed to be 4 nm to mimic the nacreous layer of Ammolite. Figure 10a–c shows the variation of reflectance for the model with different plate thicknesses. The reflectance peak changes to a longer wavelength as *d*_1_ increases. Moreover, the variation of *d*_1_ (with introducing a standard deviation) achieved broadened reflection bands (Fig. 10d–f), which are similar to real reflection from Ammolite, as shown in Fig. 2. We verified that *d*_1_ of the aragonite plates is essential for the color change from blue to red. Next, we evaluated the effect of variation of the lamination period (*d*) for the structural color. As shown in Fig. S17 in the SI, gradual variation of *d* broadens the reflectance spectra. Thus, the homogeneous period is important for the appearance of vivid colors.

**Fig. 11:**
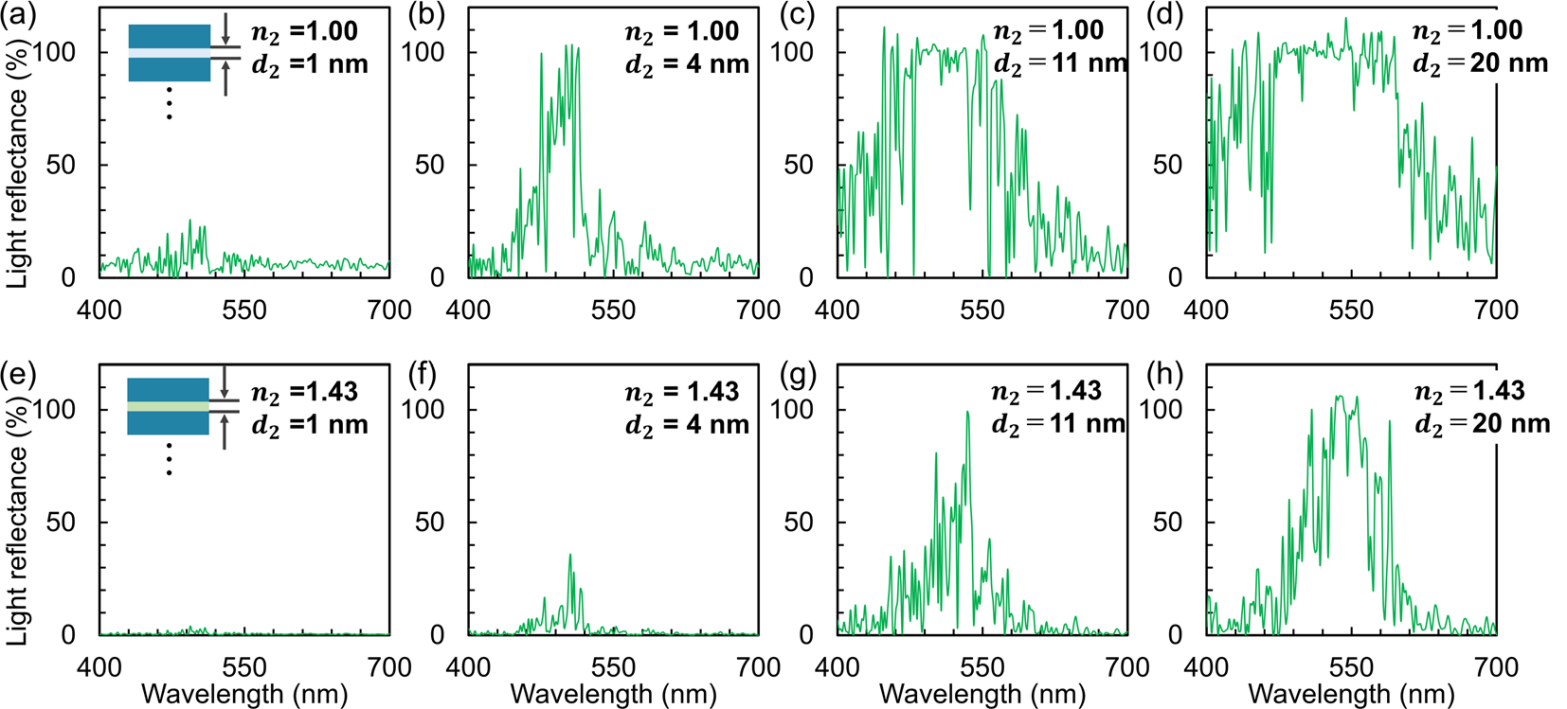
Reflectance spectra from the laminated structure having *d*_1_ of 150 nm and a standard deviation of 15 nm with different reflective indices (*n*_2_) and *d*_2_. (**a**–**d**) *n*_2_ = 1.0 with *d*_2_ = 1, 4, 11, and 20 nm. (**e**–**h**) *n*_2_ = 1.43 with *d*_2_ = 1, 4, 11, and 20 nm.

In the second step, we show the dependence of chroma on *d*_2_. Figure 11a–d shows the variation of the reflectance spectra from the nacreous layers having *d*_1_ of 150 nm and a standard deviation of 15 nm by a change in *d*_2_ with a refractive index of 1.00 (air gap). As *d*_2_ decreases from the standard value (4 nm) to 1 nm, the reflectance of the interfering wavelengths decreases (Fig. 11a). Weak reflection from the Madagascar ammonite is ascribed to the very small gap between the aragonite plates, as shown in Figs. 2c and 3f. An increase in *d*_2_ to 20 nm increases the reflection in all ranges of light as white noise (Fig. 11c, d). We found that a reflectance band that was calculated with *d*_2_ of 4 nm is similar to that of the nacreous layers of Ammolite (Fig. 11b).

To study the color saturation of abalone and nautilus, we simulated the reflectance spectra from the laminated structure having *d*_1_ of 150 nm and a standard deviation of 15 nm with a refractive index of 1.43 for organic sheets between aragonite plates. When *d*_2_ was 11 nm, the total reflection decreases with an increase in the refractive index (Fig. 11g). This indicates that abalone shells show pale blue and green due to relatively weak reflection. The reflection band broadens with an increase in *d*_2_ to 20 nm (Fig. 11h). Thus, the pearly luster of nautilus is attributed to a large gap with organic sheets in the nacreous layer. The reflectance signals of the interfering wavelengths are relatively weak when *d*_2_ is smaller than 4 nm (Fig. 11e, f). These results indicate that the nanogaps around 4 nm in the nacreous layer is important for the Ammolite’s exhibition of brilliant colors with the suppression of white noise.

The essence of vivid colors from the nacreous layers is deduced to be the small interlamellar gap (~ 4 nm) that is filled by air. We found that the reflectance decreases as the interlamellar gap decreases, which was ~ 0.1% for a gap of 4 nm at a wavelength of 500 nm, from the calculation of multiple reflections^32^ under normal incidence on the interlamellar spacing sandwiched by aragonite plates (Fig. S18 and Eq. S1 in the SI). Since the penetration depth of the incident light increases with a decrease in the reflection rate, the number of the reflection planes increases inevitably (Fig. S18a in the SI). From a theoretical calculation (Fig. S19 and Eq. S2-4 in the SI), the full-width at half maximum of the interference reflection signal decreases as the number of the reflection planes increases. Therefore, the color saturation increases with reducing the interlamellar spacing.

## Conclusion

The origin of brilliant structural colors of specific ammonite fossils—Ammolites—was clarified by the detailed characterization of the microstructures and optical properties with comparison to various kinds of nacreous layers. The stacking structures with uniform plate thicknesses in the fossilized nacreous layers of Ammolites are almost the same as those of contemporary shells. We found that the homogeneous periodicity of the laminated structures with the interlaminar nanogaps around 4 nm is essential for the exhibition of brilliant colors with the suppression of white noise. Our findings will lead to understanding of the structural color originating from multilayer interference and the development of applications to new optical devices.

### Experimental

### Materials

Samples of Ammolites (*Placenticeras meeki*, *Placenticeras costatum*, and *Placenticeras intercalare*) from the Bearpaw Formation in Alberta, Canada, ammonites (Guessing *Cleoniceras*) from Madagascar^33^, abalones (Haliotis iris) from Zealand^34–36^, and nautiluses (*Nautilus pompilius*) from the Philippines were used. We cannot identify the specific species of Placenticeras because differences in the shell shapes have not been confirmed. Fossils of Ammolite collected from the mines owned by Tracy & Beth Day Chief in Alberta, Canada, were provided by Ms. Akemi Koyama at Canada Business Service Co., Ltd. (3-17-7-2 Shukugawara, Tama-ku, Kawasaki 214-0021, Japan, https://www.canada-business.co.jp) and Mr. David Homayon. Ammonite fossils from Madagascar were also obtained from Canada Business Service. Shells of abalones and nautiluses were purchased from Takashell Co., Inc. (2-195　Nishiyuge, Yao, Osaka 581-0035,　Japan, http://www.takashell.co.jp).

### Measurements

The colors were evaluated by the CIE chromaticity diagram using image analysis software (ImageJ Fiji). The reflectance spectra were obtained by a combination of a digital microscope (VHX-970 F, Keyence) and a microchannel spectrometer (Photonic multi-channel analyzer PMA-12, Hamamatsu Photonics). White light from a light-emitting diode (LED Luminar Ace LA-HDF158A, Hayashi) projected on a white plate (polytetrafluoroethylene) was used as the reference (Fig. S6(b) in the SI). Samples were buried in epoxy resin (C&A) and polished to expose a cross section of nacreous layers. The surfaces were etched with a 5 mM ethylene diamine tetraacetate (Kanto Chemical) solution for 15 s to remove damaged topmost layers and then coated with osmium for detailed observation using a scanning electron microscope (SEM, JEOL JSM-7100) operated at 5.0 kV. EBSD measurements were carried out, after coating with osmium, on a JEOL JSM-70,001 F field-emission SEM equipped with an Oxford EBSD detector. Samples were also buried in epoxy resin and polished with colloidal silica after exposing a cross section of nacreous layers. The plate thicknesses (*d*_1_) were randomly measured (*n* = 100) with SEM images using the image analysis software. The lamination periods (*d*) were measured by acquiring the grayscale in SEM images, and the average values were calculated from the intervals of the peaks. Internal observation was performed by transmission electron microscopy (TEM, FEI Tecnai Osiris, FEI Tecnai G2) after being processed by focused ion beam (FIB, FEI Quanta 3D FEG). Elemental mappings were performed by TEM-EDS (FEI Tecnai Osiris). Thermogravimetric analysis was performed using a Shimadzu DTG-60. Nacreous layers were ground into powders and tested under flowing air from 30 to 800 °C at a heating rate of 1 °C min^− 1^.

### Optical simulation

The light reflectance of the nacres with narrow plate gaps was simulated using the software (FullWAVE FDTD Add-on - Windows / Linux, Version 2024.09, Synopsys, https://www.synopsys.com/photonic-solutions/rsoft-photonic-device-tools.html) based on tthe 2D finite-difference time-domain (2D-FDTD) method. Detailed simulation conditions are described in Fig. S16 in the SI.

### Modification of nacreous layers of the abalone for vivid coloration

In the synthesis of analogs, abalone shells were boiled in 0.5 M NaOH (Kanto Chemical) at 100 °C for 2 h in an autoclave, immersed in pure water for 24 h, and baked in air at 200 °C for 2 h to remove organic substance between the nacreous layer of abalone shells according to a previous work^37^. The removal of the interlamellar membrane was confirmed from variation of weight losses evaluated by thermogravimetric analysis in the range of 100–600 °C before (~ 14% and after (~ 3%) the treatment and TEM-EDS (Fig. S14 in the SI). The treated shells were buried in ultraviolet curable resin (A One Planning) and pressed with a hydraulic oil press at approximately 1000 MPa.

### Terminology section

**Table:** 

Term	Explanation
coleopteran	The largest order of insects comprising the beetles and weevils and being distinguished by a pair of forewings that are usually hard and rigid.
elytron	The hardened forewing of beetles (coleopterans).
saturation (chroma)	Chromatic purity. The degree of difference from the gray having the same lightness.
hue	The attribute of colors that permits them to be classed as red, yellow, green, blue, or an intermediate between any contiguous pair of these colors
Bearpaw Formation	A geologic formation of the Late Cretaceous (Campanian) age. It outcrops in the U.S. state of Montana, as well as the Canadian provinces of Alberta and Saskatchewan. It includes a wide range of marine fossils, as well as the remains of a few dinosaurs.
Commission Internationale de I’Edairage (CIE) chromaticity diagram	Mathematical models that comprise a “standard observer”, which is a static idealization of the color vision of a normal human. This diagram is a fundamental tool for measuring color for industry, including inks, dyes, and paints, illumination, color imaging, etc.
inverse pole figure (IPF) map	The map of the directional distribution of a set of reference vectors in terms of a fixed crystallographic frame.
multiple of uniform distribution (MUD) value	The value of the maximum intensity of the contoured pole figures. This value is used as a measure of crystallographic orientation distribution in pole figures.
pole figure	A two-dimensional graphical representation of orientation, showing the orientation of a selected plane normal (a pole) with respect to the sample reference frame.
overprint	A geological process that superimposes a set of characteristics (new structure, texture, or mineral composition) on rocks, including fossils.
Bragg-Snell equation	The equation of applying Bragg’s law of diffraction and Snell’s law of refraction. This equation describes the reflection maximum (transmission minimum), which varies with the angle of observation, lamination period or material composition.
Bragg-Snell reflection	The reflection of which the light interferes in accordance with the Bragg-Snell equation.

## Supplementary Information

Below is the link to the electronic supplementary material.

Supplementary Material 1

## Data Availability

The authors declare that the data supporting the findings of this study are available within the paper and its Supplementary Information file. Characterised fossils are deposited in a repository of Materials Chemistry Laboratory at Keio University. Raw data files and the specimens (reference codes: MCL-am001b, MCL-am002g, MCL-am003r, MCL-am004m) are available from the corresponding author upon reasonable request for replicating or confirming the results.
