# Supplementary material for: Brilliant structural colors originating from reflection by nanogaps of nacreous layers in fossilized ammonite shells
Source: Sci Rep. 2025 Oct 30;15:37541. doi: 10.1038/s41598-025-21872-z (PMC12575706; doi:10.1038/s41598-025-21872-z)
Supplement: Supplementary file 1 — Supplementary Material 1 [file 41598_2025_21872_MOESM1_ESM.docx]

**Supporting Information for**

**Brilliant structural colors originating from reflection by nanogaps of nacreous layers in fossilized ammonite shells**

Naoki Hizukuri^1^, Yuri Oshima^1^, Yuta Yagi^1^, Hayato Sasazawa^1^, Yuya Oaki^1^, Hiroyuki Tsuda^1^, Hiroaki Imai^1 *^

^1^ School of Integrated Design Engineering, Faculty of Science and Technology, Keio University, 3-14-1 Hiyoshi, Kohoku-ku, Yokohama 223-8522, Japan /^*^E-mail: hiroaki@applc.keio.ac.jp

Fig. S1 Photos of a whole shell and a green piece of Ammolite and an elytron of a jewel beetle and their reflectance spectra.

Fig. S2 Photos of blue, green, and red pieces of Ammolite fossils, an abalone shell, an ammonite fossil from Madagascar and a nautilus shell.

Fig. S3 Photos of powders obtained by grinding of an Ammolite fossil.

Fig. S4 Weigh loss of pieces of Ammolite and an abalone shell evaluated by thermogravimetry.

Fig. S5 Photos and reflectance spectra from blue-green and orange pieces of Ammolite.

Fig. S6 Schematic illustrations of the experimental setup and a typical reflectance spectrum.

Fig. S7 The angle dependence of colors and reflectance spectra of red, green, blue pieces of Ammolite.

Fig. S8 Pole figures showing crystallographic orientations of the grains in Fig. 4.

Fig. S9 Principle of reflective structural coloration obtained from multilayers.

Fig. S10 Photos and cross-sectional SEM images of various samples to evaluate the depth profiles of the lamination periods.

Fig. S11 Photos, cross-sectional SEM images, the depth profiles of the lamination periods, and enlarged SEM images of the cross sections of a high-quality green piece of Ammolite.

Fig. S12 Photos, cross-sectional SEM images, and the depth profiles of the lamination periods of a pale orange piece of Ammolite.

Fig. S13 Photos indicating color change with polishing.

Fig. S14 A high-angle annular dark-field (HAADF) image with TEM-EDS elemental mapping images for an FIB-cut sample of the nacreous layer of an abalone shell after the treatment.

Fig. S15 Cross-sectional SEM images of blue, blue-green, green, orange, red pieces of Ammolite.

Fig. S16 A schematic model for the 2-dimensional finite-difference time-domain (2D-FDTD) method.

Fig. S17 Simulated reflectance spectra from the periodic structures with a gradual variation of the plate thickness.

Fig. S18 A schematic illustration of multiple reflections under normal incidence on a single air spacing sandwiched by aragonite plates, and the calculated total reflectance as a function of the interlamellar gap distance.

Fig. S19 Schematic illustrations of multilayer interference and the width of a reflectance spectrum.


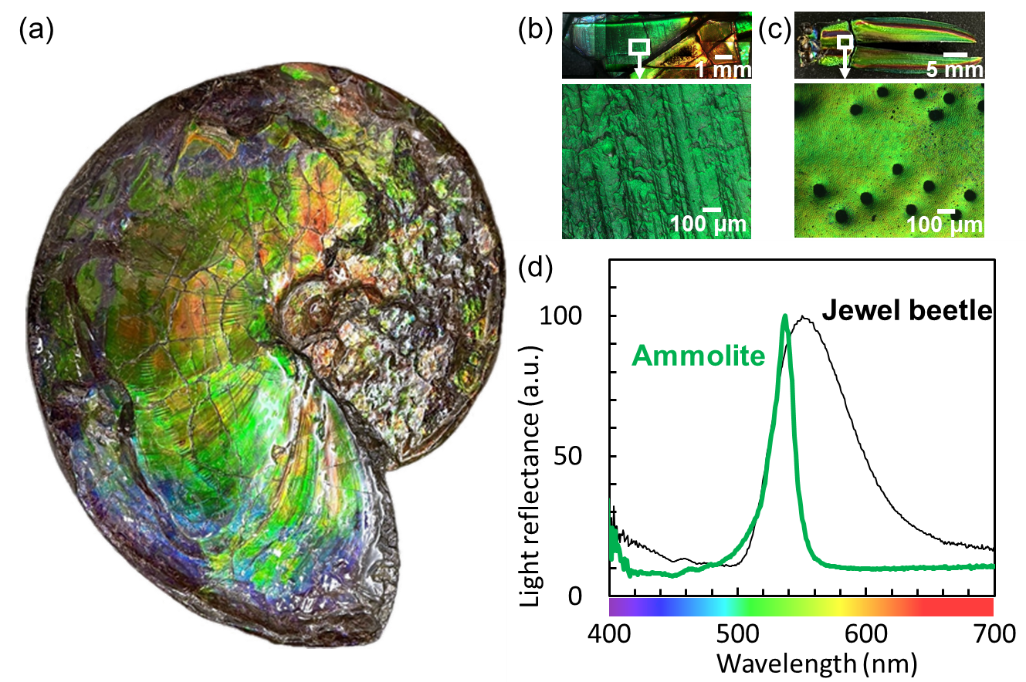


Fig. S1 Photos of (a) a whole shell of Ammolite (*Placenticeras meeki*), (b) a green piece of Ammolite, and (c) an elytron of a jewel beetle (*Chrysochroa fulgidissima*) and (e) their reflectance spectra.


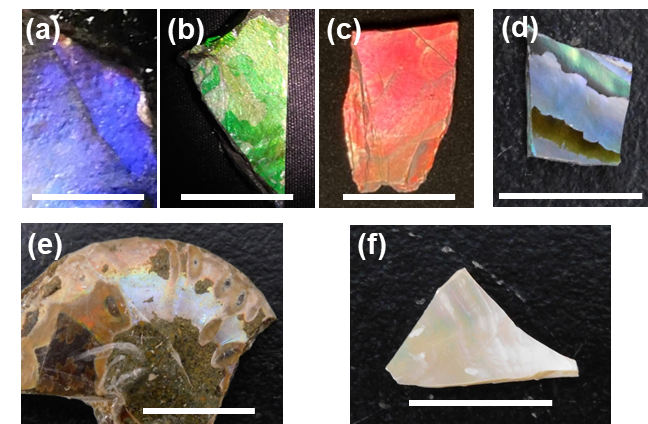


Fig. S2 Photos of (a) blue, (b) green, and (c) red pieces of Ammolite fossils, (d) an abalone shell from New Zealand, (e) an ammonite fossil from Madagascar and (f) a nautilus shell from Philippines. Scale bars: 10 mm.

(a) (b)


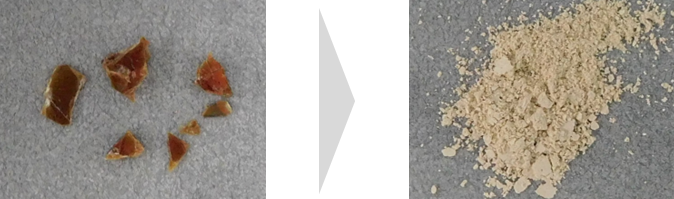

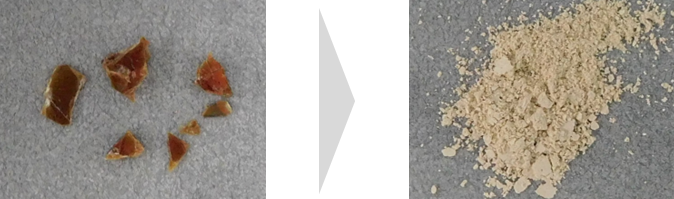


Fig. S3 Photos of red pieces of Ammolite (a) before and (b) after grinding.


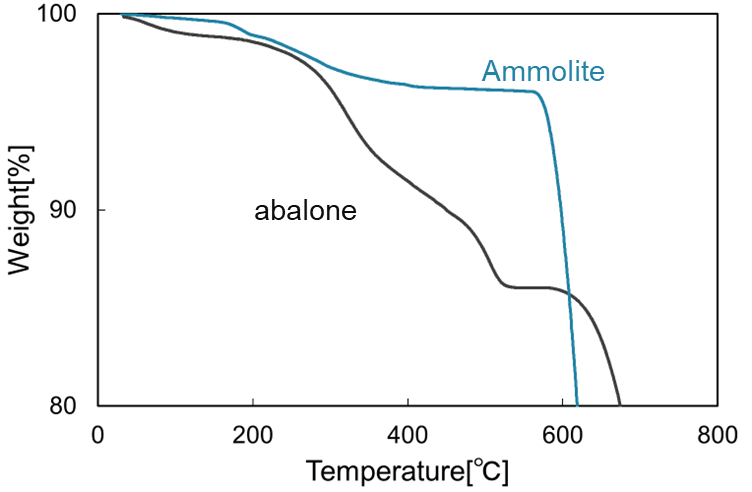


Fig. S4 Weigh loss of pieces of Ammolite and an abalone shell evaluated by thermogravimetric analysis. The organic contents in Ammolite and abalone shells were estimated to be 2–3 and ∼10 wt%, respectively, from the weight loss in the range of 200 and 550°C.


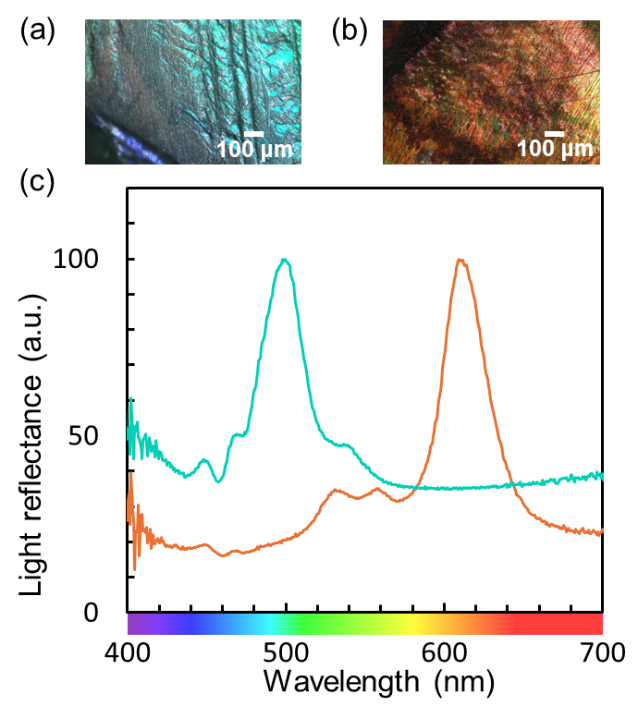


Fig. S5 (a, b) Photos and (c) reflectance spectra from (a) blue-green and (b) orange pieces of Ammolite.


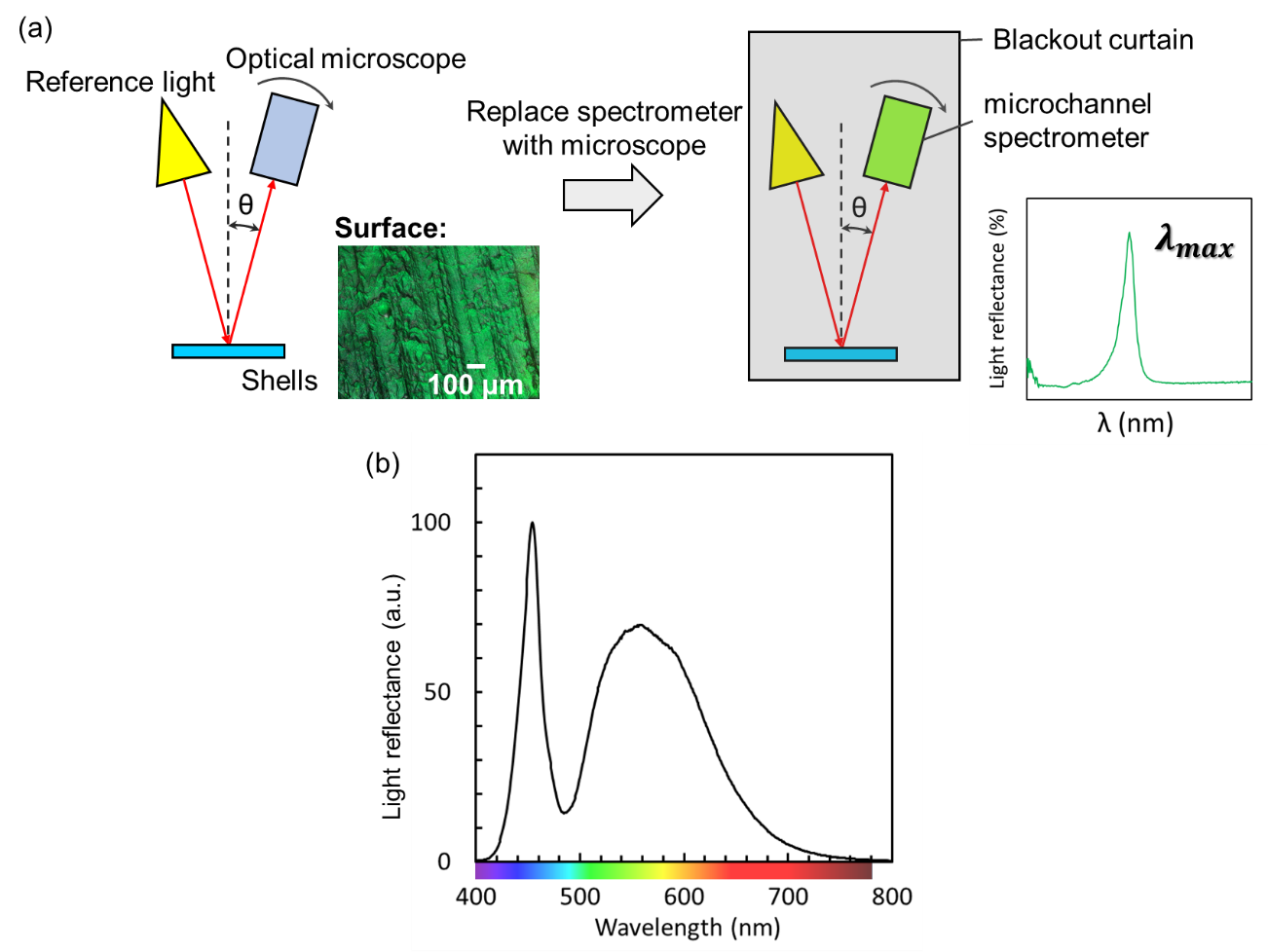


Fig. S6 (a) Schematic illustrations of the experimental setup for the angle dependence of colors and reflectance spectra. (b) A typical reflectance spectrum of the reference light irradiated on a white polytetrafluoroethylene plate.


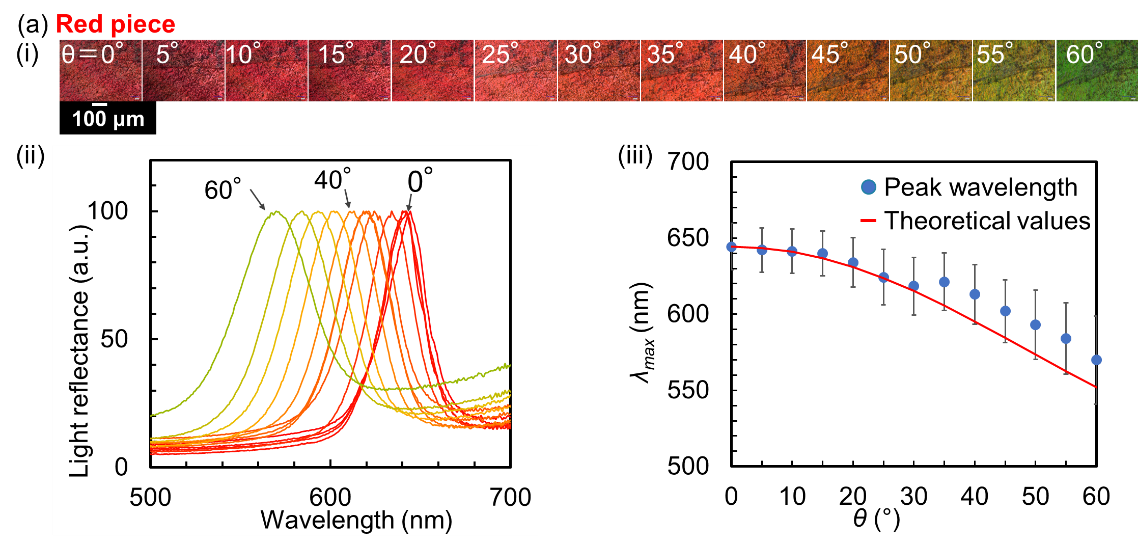


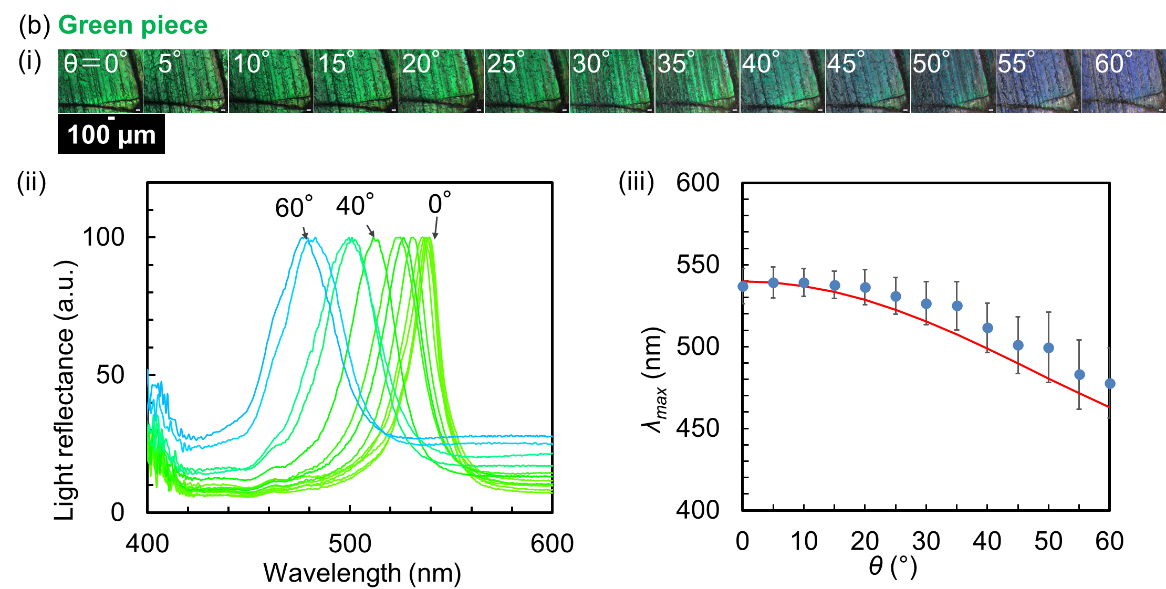


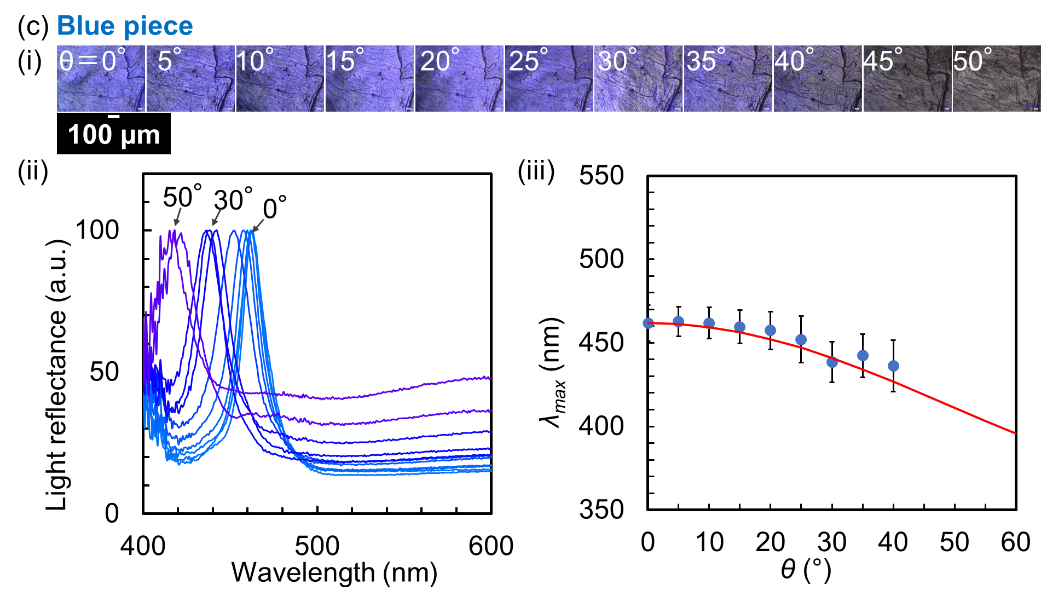


Fig. S7 The angle dependence of colors and reflectance spectra of (a) red, (b) green, (c) blue pieces of Ammolite. (i) Photos of surface, (ii) reflectance spectra, and (iii) the relationship between the observation angle (*θ*) and the peak wavelength (*λ_max_*) of the spectra. Error bars indicate the half-width values of the peaks. The theoretical values calculated using Eq. (1) are shown as red curves.


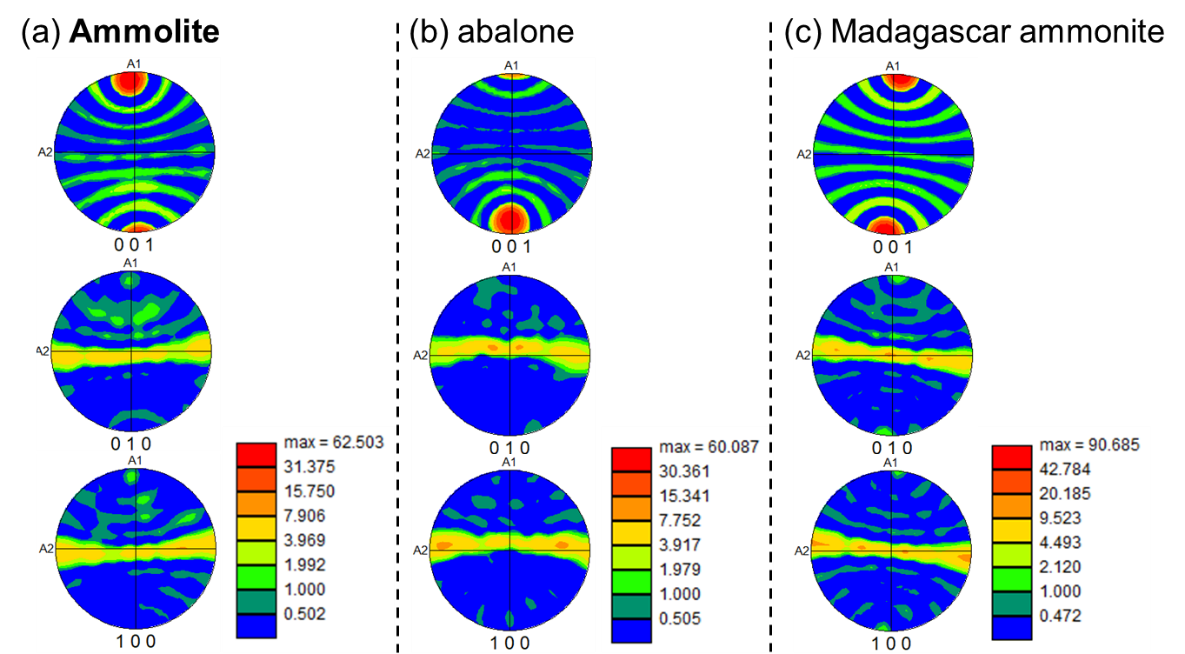


Fig. S8 Pole figures showing crystallographic orientations of the grains in Fig. 4. (a) Ammolite (green), (b) abalone (blue), and (c) Madagascar ammonite (blue). The location of red parts for 001 mean that the *c* axis is perpendicular to the nacre surface of all the samples. The lateral yellow bands for 010 and 100 suggest that the *a* and *b* axes of aragonite tablets rotate along the *c* axis in the nacreous layer of all the samples.


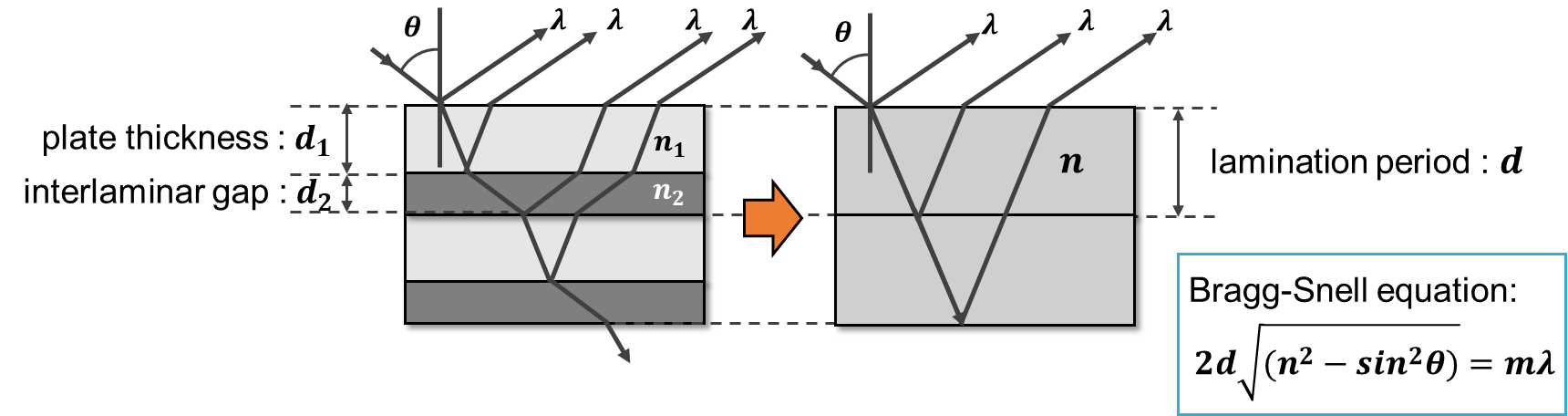


Fig. S9 Principle of reflective structural coloration obtained from multilayers.


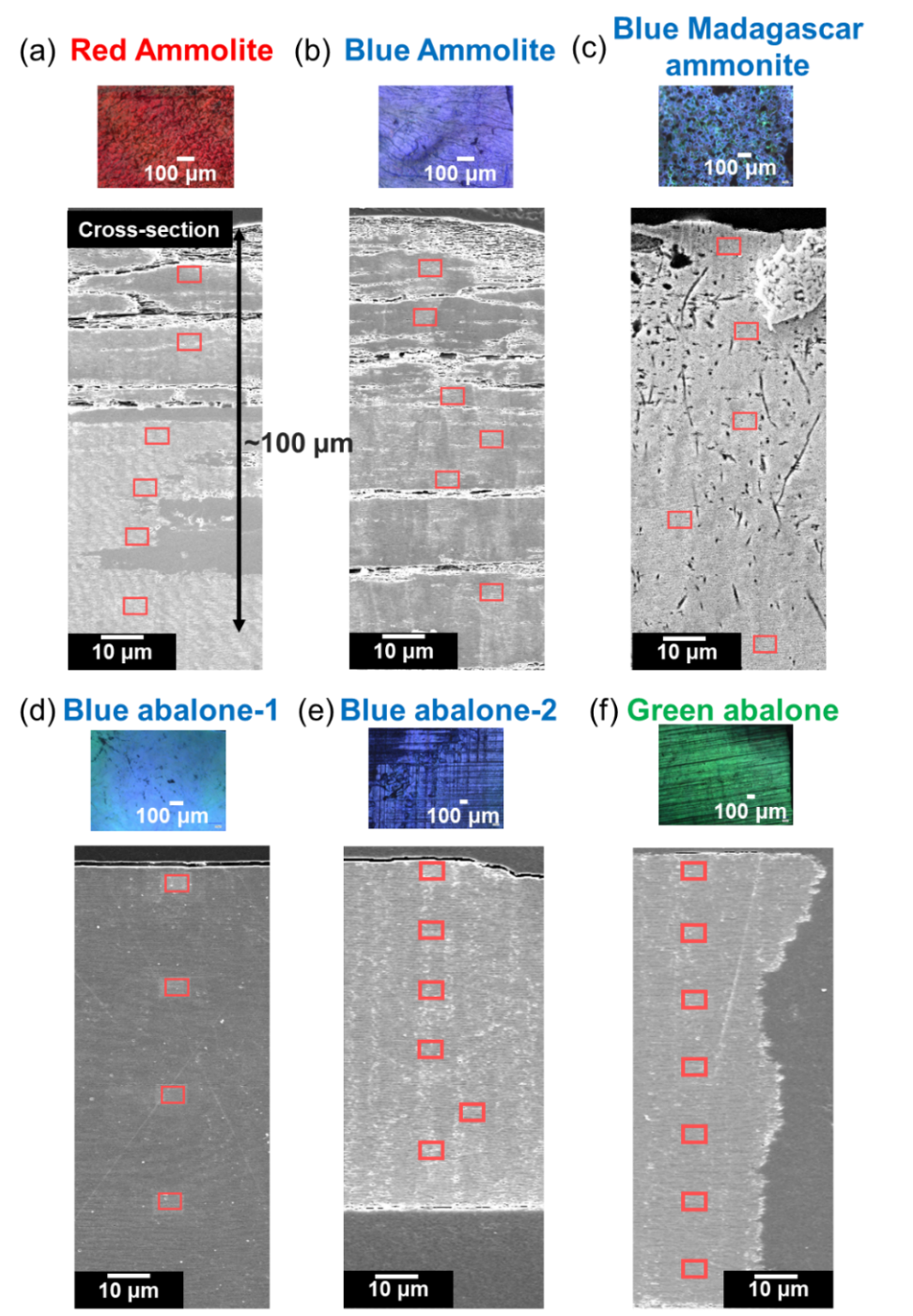


m=2

$$\boldsymbol{2}\boldsymbol{nd=m\lambda}$$

m=1

Fig. S10 Photos and cross-sectional SEM images of various samples to evaluate the lamination periods that depend on the depth from the surface. (a) Red and (b) blue pieces of Ammolite; (c) a blue piece of Madagascar ammonite; (d, e) blue and (f) green pieces of abalone shells.


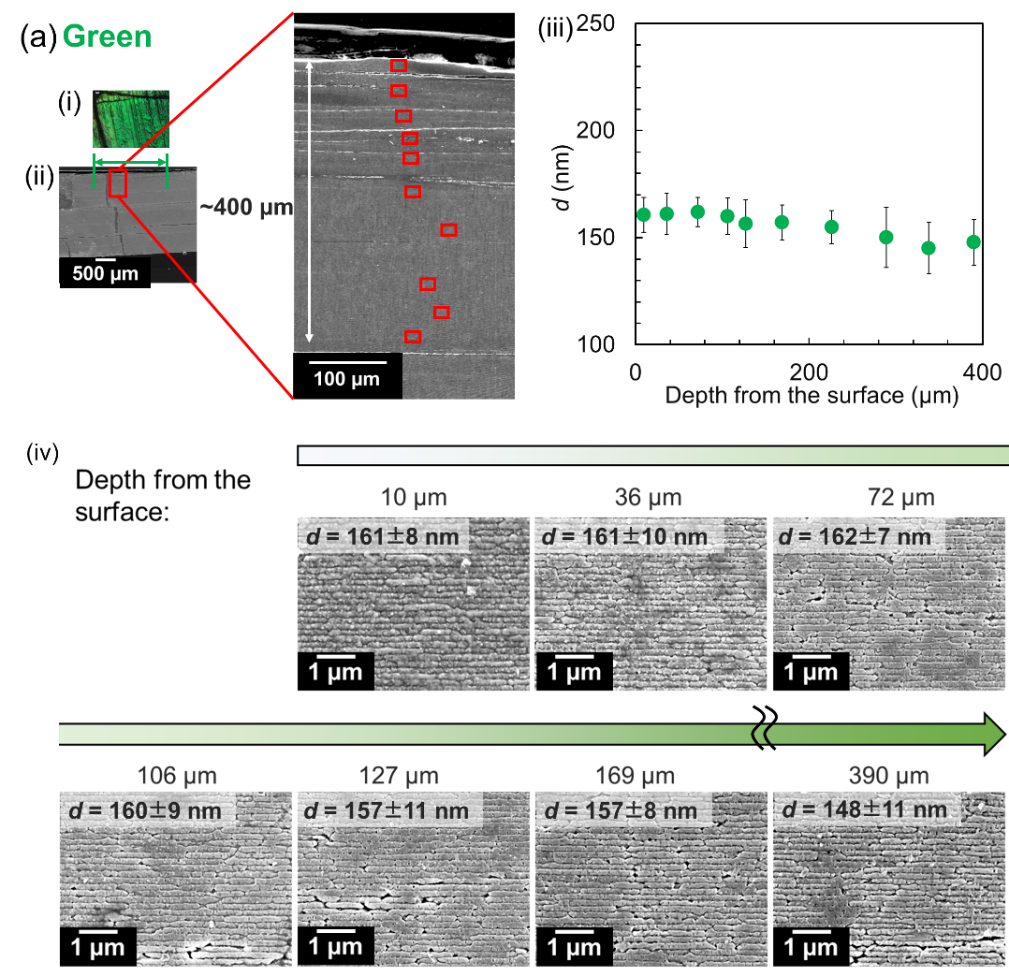


Fig. S11 (i) Photos, (ii) cross-sectional SEM images, and (iii) the depth profiles of the lamination periods (*d*) of a high-quality green piece of Ammolite. (iv) Enlarged SEM images of the cross sections indicated in (ii).


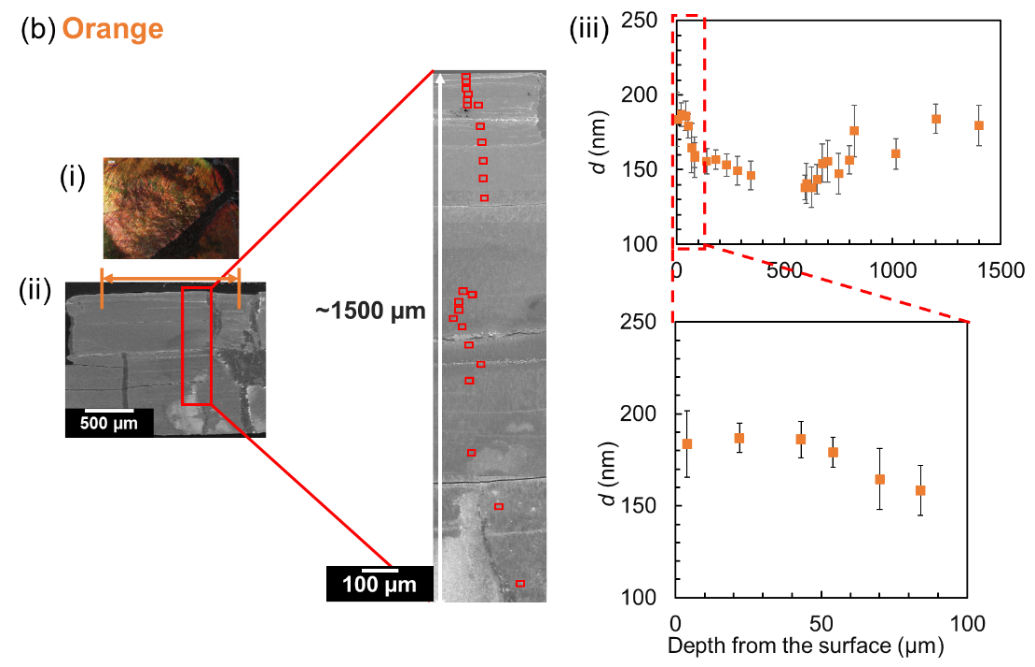


Fig. S12 (i) Photos, (ii) cross-sectional SEM images, and (iii) the depth profiles of the lamination periods (*d*) of a pale orange piece of Ammolite.


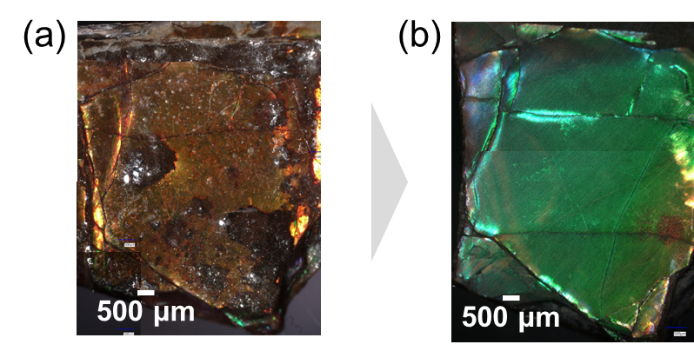


Fig. S13 Photos of color change from (a) a red piece of Ammolite to (b) green by polishing.


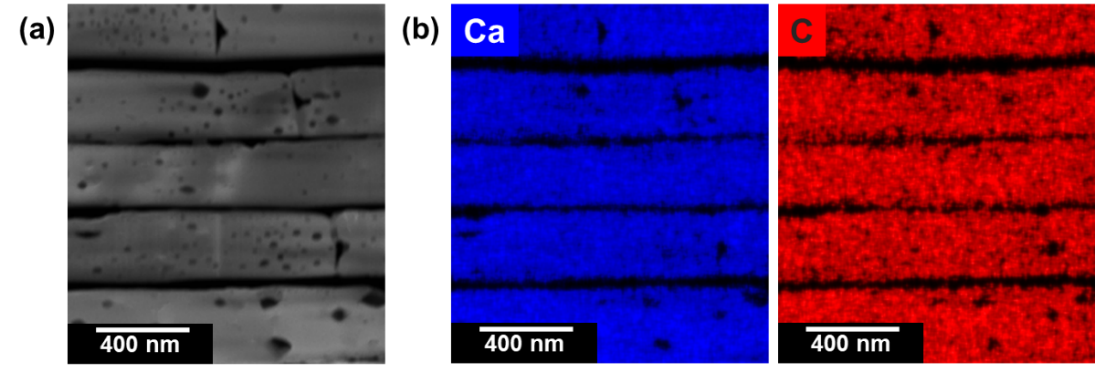


Fig. S14 A high-angle annular dark-field (HAADF) image with elemental mapping images for an FIB-cut sample of the nacreous layer of an abalone shell after the treatment (boiling in 0.5 M NaOH at 100°C for 2 h and baking in air at 200°C for 2 h). (a) A HAADF image and (b) the elemental maps of calcium and carbon. The nacreous plates are indicated by the presence of calcium. The removal of organic matter from the interlamellar gaps is confirmed by the absence of carbon.


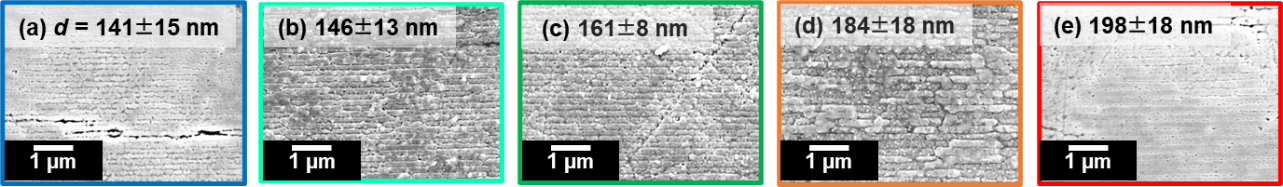


Fig. S15 Cross-sectional SEM images of (a) blue, (b) blue-green, (c) green, (d) orange, (e) red pieces of Ammolite.


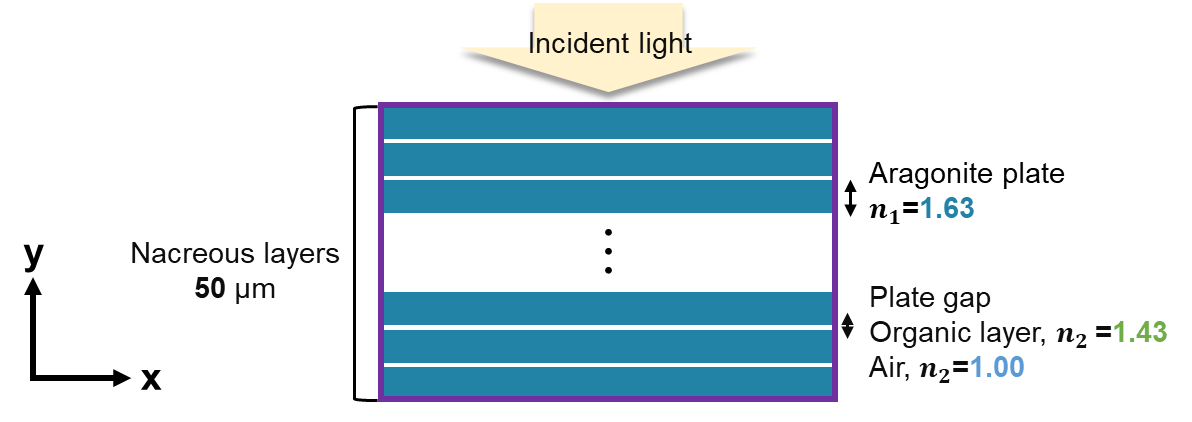


Fig. S16 A schematic model for the two-dimensional finite-difference time-domain (2D-FDTD) method using the software (FullWAVE FDTD Add-on - Windows / Linux, Version 2024.09, Synopsys, <https://www.synopsys.com/photonic-solutions/rsoft-photonic-device-tools.html>). We made two-dimensional models with the periodic boundary condition in the x direction and the absorbing, perfectly matched layer (PML) boundary condition in the y direction for nacreous layers. The refractive index of aragonite crystals was set to *n*_1_=1.63^1,2^ and the refractive index between plates was set in two ways: *n*_2_=1.43 ^1,2^ (organic layer) and *n*_2_ = 1.00 (air layer).


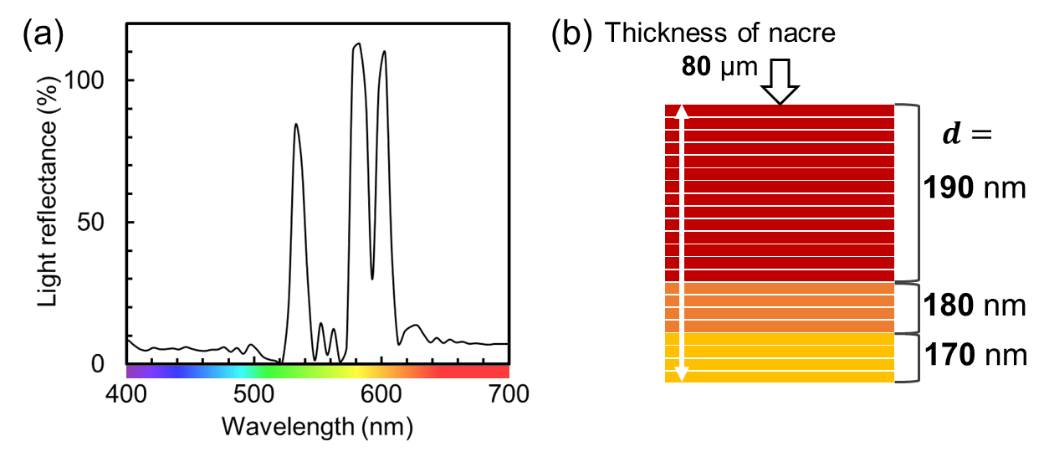


Fig. S17 (a) Simulated reflectance spectra from the periodic structures with (b) a gradual variation of the plate thickness from 170 to 190 nm and the interlaminar gap 5 nm.


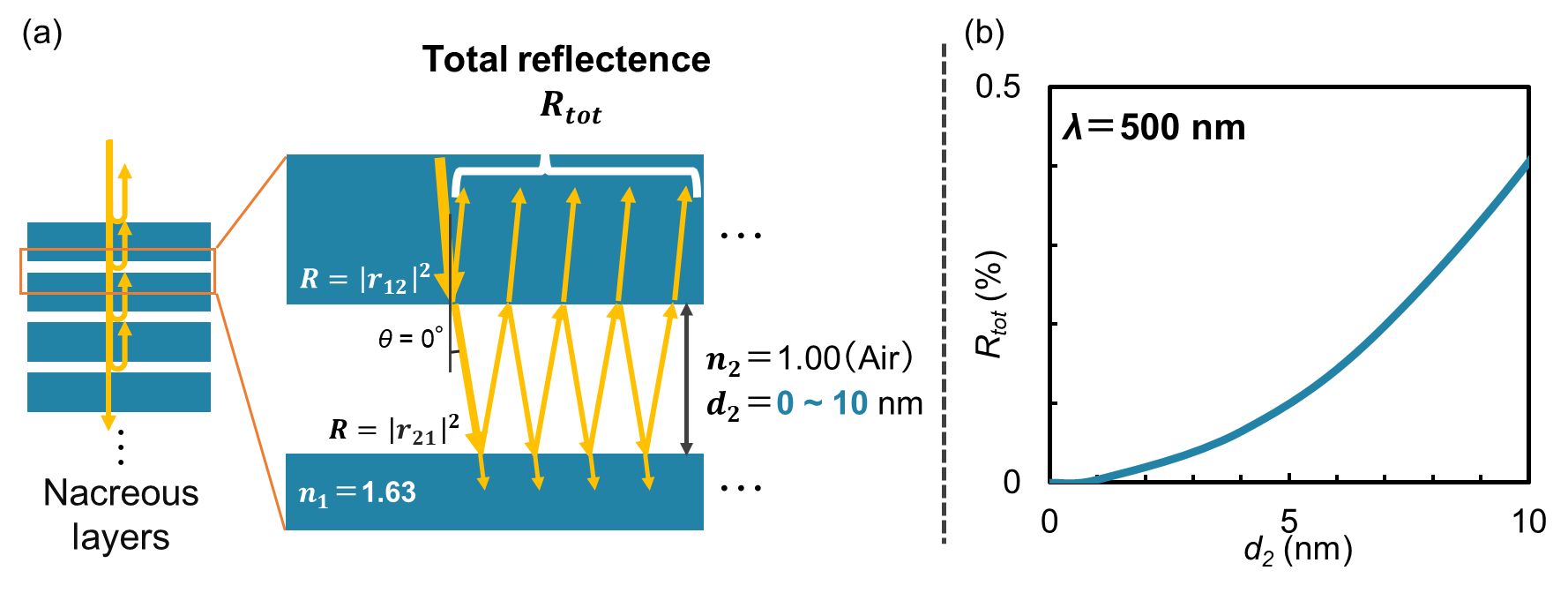


Fig. S18 (a) A schematic illustrations of multiple reflections under normal incidence on the interlamellar spacing sandwiched by aragonite plates and (b) the total reflectance ($R_{tot}$) as a function of the interlamellar gap distance (*d_2_*) calculated by Eq. S1 at a wavelength of 500 nm.

The value of $R_{tot}$ by multiple reflections^3^ on the interlamellar spacing sandwiched by aragonite plates under normal incidence (*θ* = 0°) is expressed by

$R_{tot}=\frac{F \sin^{2} \delta}{1+F \sin^{2} \delta}$ (Eq. S1),

where $\delta$: the optical phase thickness of the interlamellar spacing and *F*: the finesse coefficient.

The values of $\delta$ and *F* are calculated by

$$\delta=\frac{2\pi}{\lambda}n_{2}d_{2}$$

$$F=\frac{4R}{{(1-R)}^{2}}$$

with the reflectance (*R*) and the amplitude reflection coefficients at the aragonite/air and air/aragonite interfaces (*r*_12_ and *r*_21_, respectively), the refractive indices of the nacreous layer (*n*_1_=1.63^2^) and air (*n*_2_ = 1.00), and the wavelength of the incident light ($\lambda=500$nm).

The value of *R* is expressed by

$$R=\left| \left. r_{12} \right| \right.^{2}=\left| \left. r_{21} \right| \right.^{2}$$

$r_{12}=\frac{n_{1}-n_{2}}{n_{1}+n_{2}}$, $r_{21}=\frac{n_{2}-n_{1}}{n_{2}+n_{1}}$.


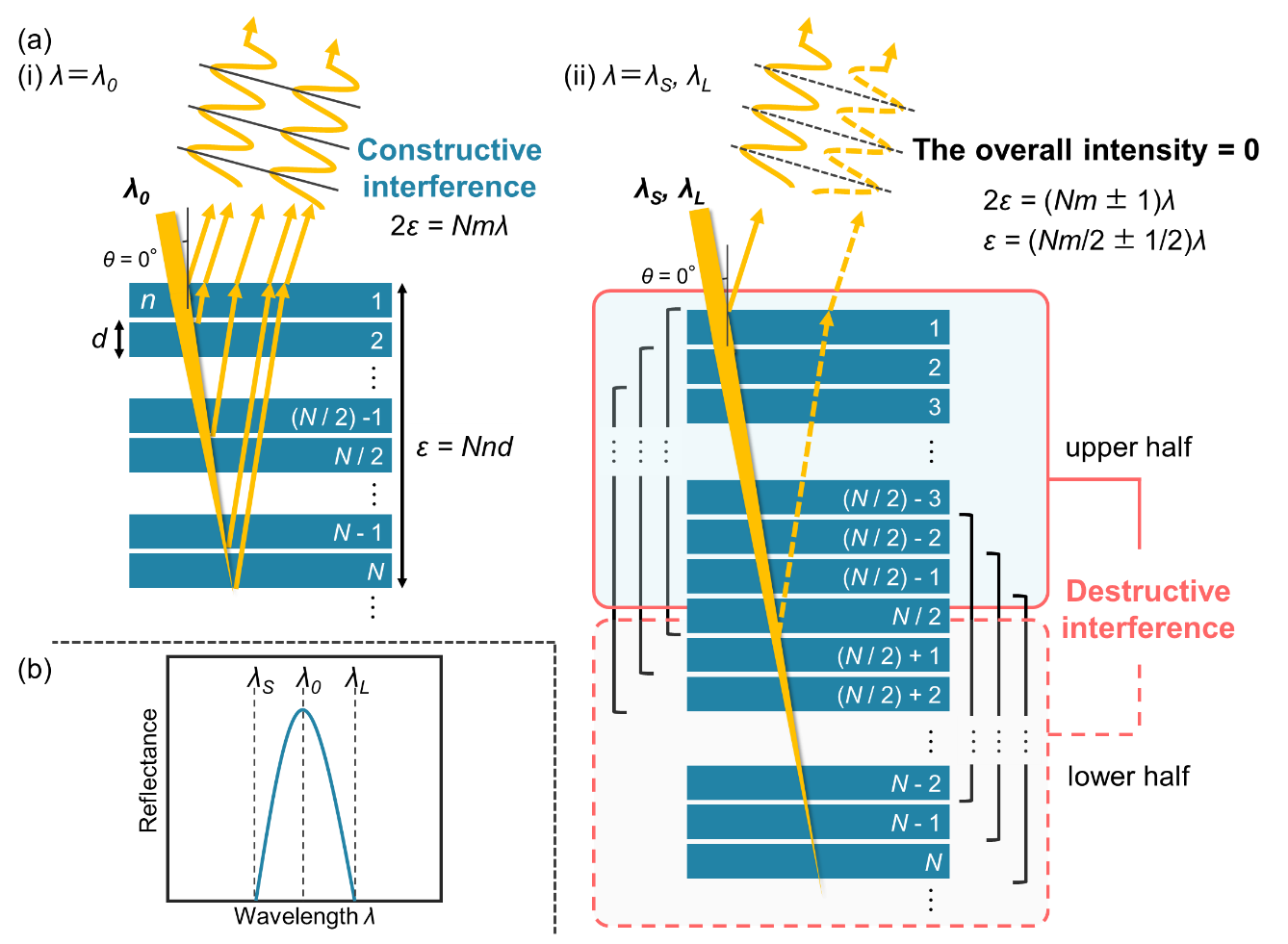


Fig. S19 (a) Schematic illustrations of multilayer interference by incident light with a wavelength of (i) *λ*₀ and (ii) *λ*_L_ or *λ*_S_ and (b) the width of a reflectance spectrum with *λ*_0_, *λ*_L_ and *λ*_S_.

The Bragg's equation for normal incidence (*θ* = 0°) is expressed by

$2nd=m\lambda$　　　　　　　　 　(Eq. S2),

where *n*: the effective refractive index, *d*: the layer distance, *m*: the reflection number, and *λ*: the wavelength.

The reflection intensity is enhanced through constructive interference at *λ = λ*₀ (Panel a-i).

Eq. 2 changes into

$2\varepsilon=Nm\lambda_{0}$ (Eq. S3)

where the optical thickness $\varepsilon=Nnd$ with the layer number (*N*).

As the incident wavelength (*λ*_S_ ≦ *λ* ≦ *λ*_L_) deviates from *λ*₀, the reflectance decreases due to misalignment of the phases of the light reflected from each boundary. When the optical path difference between the top plane of the 1st layer and the bottom plane of the *N*th layer ($2\varepsilon$) reaches (*Nm*±1) *λ* (Panel a-ii), the optical path difference between the top plane of the 1st layer and the bottom of the *N*/2th layer$(\varepsilon)$ is (*Nm* ⁄ 2±1 ⁄ 2) *λ*. In these cases, the reflected lights from the two planes annihilate each other with destructive interference. Since the destructive interference occurs between the lights reflected in the upper and lower halves of the multilayer, the overall intensity of the reflection is 0. Thus, largest and smallest wavelengths for reflection through the multilayer interference is expressed by

$2\varepsilon=(Nm-1)\lambda_{L}$.

$2\varepsilon=(Nm+1)\lambda_{S}$.

The spectral width (Panel b) is expressed by,

$$\lambda_{L}-\lambda_{S}=\frac{2\varepsilon}{Nm-1}-\frac{2\varepsilon}{Nm+1}$$

　　　　　　　　　　　 　$=\frac{2Nm\lambda}{N^{2}m^{2}-1}$. 　　　　　　　　　　(Eq. S4)

This indicates that the spectral width narrows as the number of layers *N* increases.

1. J. Salman, C. A. Stifler, A. Shahsafi, C.-Y. Sun, S. Weibel, M. Frising, B. E. Rubio-Perez, Y. Xiao, C. Draves, R. A. Wambold, Z. Yu, D. C. Bradley, G. Kemeny, P. U. P. A. Gilbert and M. A. Kats, Hyperspectral interference tomography of nacre. *Proc. Natl. Acad. Sci. USA*, **118**, 1–6 (2021).

2. M. R. Snow, A. Pring, P. Self, D. Losic and J. Shapter, The origin of the color of pearls in iridescence from nano-composite structures of the nacre. *Am. Mineral.* **89**, 1353–1358 (2004).

3. Kuroda, K. Physical optics: Propagation of light waves in media (in Japanese). *Asakura Publishing Co., Ltd., Tokyo* (2011).
